# Correction: Blood pressure monitoring in high risk pregnancy to improve the detection and monitoring of hypertension (The BUMP 1&2 trials): protocol for two linked randomised controlled trials

**DOI:** 10.1136/bmjopen-2019-034593corr1

**Published:** 2020-08-27

Dougall G, Franssen M, Tucker KL, *et al*. Blood pressure monitoring in high-risk pregnancy to improve the detection and monitoring of hypertension (the BUMP ^1^ and ^2^ trials): protocol for two linked randomised controlled trials. *BMJ Open* 2020;10:e034593. doi: 10.1136/bmjopen-2019-034593

The article has been corrected since it was published online. The affiliation of Lucy Mackillop has been updated both in author byline and Acknowledgement section.

