# Supplementary material for: Blood pressure monitoring in high-risk pregnancy to improve the detection and monitoring of hypertension (the BUMP 1 and 2 trials): protocol for two linked randomised controlled trials
Source: BMJ Open. 2020 Jan 23;10(1):e034593. doi: 10.1136/bmjopen-2019-034593 (PMC7044851; doi:10.1136/bmjopen-2019-034593)
Supplement: Author's manuscript [file bmjopen-2019-034593.draft_revisions.pdf]

BMJ Open is committed to open peer review. As part of this commitment we make the peer review history of every article we publish publicly available.

When an article is published we post the peer reviewers' comments and the authors' responses online. We also post the versions of the paper that were used during peer review. These are the versions that the peer review comments apply to.

The versions of the paper that follow are the versions that were submitted during the peer review process. They are not the versions of record or the final published versions. They should not be cited or distributed as the published version of this manuscript.

BMJ Open is an open access journal and the full, final, typeset and author-corrected version of record of the manuscript is available on our site with no access controls, subscription charges or pay-per-view fees (http://bmjopen.bmj.com).

If you have any questions on BMJ Open's open peer review process please email info.bmjopen@bmj.com

BMJ Open

Blood pressure monitoring in high risk pregnancy to improve the detection and monitoring of hypertension (The BUMP 1&2 trials): protocol for two linked randomised controlled trials.

Journal:	*BMJ Open*
Manuscript ID	bmjopen-2019-034593
Article Type:	Protocol
Date Submitted by the Author:	27-Sep-2019
Complete List of Authors:	Dougall, Greig; University of Oxford, Nuffield department of Primary Care Health Sciences<br>Franssen, Marloes; University of Oxford, Nuffield department of Primary Care Health Sciences<br>Tucker, Katherine; University of Oxford, Nuffield Department of Primary Care<br>Yu, Ly-Mee; University of Oxford, Department of Primary Care Health Sciences<br>Hinton, Lisa; University of Oxford, Health Experiences Research Group, Nuffield Department of Primary Care Health Sciencs<br>Rivero-Arias, Oliver; University of Oxford, Nuffield Department of Population Health<br>Abel, Lucy; University of Oxford<br>Allen, Julie; University of Oxford, Nuffield Department of Primary Care Health Sciences<br>Band, Rebecca; University of Southampton, Psychology<br>Chisholm, Alison; University of Oxford Nuffield Department of Population Health, Maternal Health and Care<br>Crawford, Carole; University of Oxford, Nuffield department of Primary Care Health Sciences<br>Green, Marcus; Action on Pre-eclampsia<br>Greenfield, Sheila; University of Birmingham, Primary Care Clinical Sciences<br>Hodgkinson, James ; University of Birmingham, Primary Care Clinical Sciences, Institute of Applied Health Research<br>Leeson, Paul; University of Oxford, Division of Cardiovascular Medicine<br>McCourt, Christine; Department of Midwifery and Child health, City University London<br>MacKillop, Lucy; Oxford University Hospitals NHS Trust, Nuffield Department of Obstetrics & Gynaecology<br>Nickless, Alecia; University of Oxford, Nuffield Department of Primary Care Health Sciences<br>Sandall, Jane; Kings College, London, Health and Social Care Research Division<br>Santos, Mauro; University of Oxford, Institute of Biomedical Engineering, Department of Engineering Science<br>Tarassenko, Lionel; University of Pittsburgh, Engineering Science<br>Velardo, Carmelo; University of Oxford, Institute of Biomedical

Engineering
Wilson, Hannah; King’s College London, Women’s Health Academic Centre
Yardley, Lucy; University of Southampton, Psychology; University of Bristol, School of Health Sciences
Chappell, Dr Lucy; King’s College London, Women’s Health Academic Centre
McManus, Richard; University of Oxford, Dept of Primary Care Health Sciences

Keywords: Pregnancy, Gestational Hypertension, Pre-eclampsia, Hypertension < CARDIOLOGY, Self-Monitoring, Blood Pressure

SCHOLARONE™
Manuscripts

I, the Submitting Author has the right to grant and does grant on behalf of all authors of the Work (as defined in the below author licence), an exclusive licence and/or a non-exclusive licence for contributions from authors who are: i) UK Crown employees; ii) where BMJ has agreed a CC-BY licence shall apply, and/or iii) in accordance with the terms applicable for US Federal Government officers or employees acting as part of their official duties; on a worldwide, perpetual, irrevocable, royalty-free basis to BMJ Publishing Group Ltd ("BMJ") its licensees and where the relevant Journal is co-owned by BMJ to the co-owners of the Journal, to publish the Work in this journal and any other BMJ products and to exploit all rights, as set out in our licence.

The Submitting Author accepts and understands that any supply made under these terms is made by BMJ to the Submitting Author unless you are acting as an employee on behalf of your employer or a postgraduate student of an affiliated institution which is paying any applicable article publishing charge ("APC") for Open Access articles. Where the Submitting Author wishes to make the Work available on an Open Access basis (and intends to pay the relevant APC), the terms of reuse of such Open Access shall be governed by a Creative Commons licence – details of these licences and which Creative Commons licence will apply to this Work are set out in our licence referred to above.

Other than as permitted in any relevant BMJ Author's Self Archiving Policies, I confirm this Work has not been accepted for publication elsewhere, is not being considered for publication elsewhere and does not duplicate material already published. I confirm all authors consent to publication of this Work and authorise the granting of this licence.

For peer review only

Blood pressure monitoring in high risk pregnancy to improve the detection and monitoring of hypertension (The BUMP 1&2 trials): protocol for two linked randomised controlled trials.

*Greig Dougall[1], *Marloes Franssen[1], Katherine L Tucker[1], Ly-Mee Yu,[1] Lisa Hinton, [1] Oliver Rivero-Arias [2], Lucy Abel[1], Julie Allen[1], Rebecca J Band[10], Alison Chisholm[1], Carole Crawford[1], Marcus Green[3], Sheila Greenfield[4], James Hodgkinson[4], Paul Leeson[5], Christine McCourt[6], Lucy Mackillop[7], Alecia Nickless[1], Jane Sandall[8], Mauro Santos[9], Lionel Tarassenko[9], Carmelo Velardo[9], Hannah Wilson[8], Lucy Yardley[10], [+]Lucy C Chappell[8], and [+]Richard J McManus[1]

* Joint First Authors

[+] Joint Senior Authors

[1] Nuffield Department of Primary Care Health Sciences, University of Oxford, Oxford.
[2] National Perinatal Epidemiology Unit (NPEU), Nuffield Department of Population Health, University of Oxford, Oxford.
[3] Action on Pre-eclampsia, The Stables, 80 B High Street, Evesham, Worcestershire.
[4] Institute of Applied Health Research, University of Birmingham, Birmingham.
[5] Cardiovascular Clinical Research Facility, RDM Division of Cardiovascular Medicine, University of Oxford.
[6] Centre for Maternal & Child Health Research, School of Health Sciences, City, University of London, London.
[7] Nuffield Department of Women's & Reproductive Health, University of Oxford, Oxford.
[8] Department of Women and Children's Health, King's College London, St Thomas' Hospital, London.
[9] Institute of Biomedical Engineering, Department of Engineering Science, University of Oxford, Oxford.
[10] Department of Psychology, University of Southampton, Southampton, UK and School of Psychological Science, University of Bristol, Bristol.

Corresponding author: Prof Lucy Chappell

Email: lucy.chappell@kcl.ac.uk

Telephone: +44 (0) 7973 855025

Address: Department of Women and Childrens' Health. King's College London.

St Thomas' Hospital, Westminster Bridge Road, London. SE1 7EH.

Word count: 5147 Intro to Discussion (excluding title page, abstract, references, tables and figures)

Number of tables: 4 **Number of figures:** 5

For peer review only

For peer review only - http://bmjopen.bmj.com/site/about/guidelines.xhtml

ABSTRACT

Introduction:

Self-monitoring of blood pressure (BP) in pregnancy could improve the detection and management of pregnancy hypertension, whilst also empowering and engaging women in their own care. Two linked trials aim to evaluate whether BP self-monitoring in pregnancy improves the detection of raised BP during higher risk pregnancies (BUMP 1) and whether self-monitoring reduces systolic BP during hypertensive pregnancy (BUMP 2).

Methods and analyses:

Both are multicentre, non-masked, parallel group, randomised controlled trials. Participants will be randomised to self-monitoring with telemonitoring or usual care. BUMP 1 will recruit a minimum of 2262 pregnant women at higher risk of pregnancy hypertension and BUMP 2 will recruit a minimum of 512 pregnant women with either gestational or chronic hypertension. The BUMP 1 primary outcome is the time to the first recording of raised BP by a healthcare professional. The BUMP 2 primary outcome is mean systolic BP between baseline and delivery recorded by healthcare professionals. Other outcomes will include maternal and perinatal outcomes, quality of life, and adverse events. An economic evaluation of BP self-monitoring in addition to usual care compared to usual care alone will be assessed across both study populations within trial and with modelling to estimate long-term cost-effectiveness. A linked process evaluation will combine quantitative and qualitative data to examine how BP self-monitoring in pregnancy is implemented and accepted in both daily life and routine clinical practice.

Ethics and Dissemination:

The trials have been approved by a Research Ethics Committee (17/WM/0241) and relevant research authorities. They will be published in peer-reviewed journals and presented at national and international conferences.

Discussion:

The BUMP trials will assess the effectiveness and cost-effectiveness of BP self-monitoring during higher risk and hypertensive pregnancies. If shown to be effective, BP self-monitoring would be applicable to a large population of pregnant women.

Trial Registration: NCT03334149 (clinicaltrials.gov)

Word Count: 296

Keywords: Pregnancy, Gestational Hypertension, Pre-eclampsia, Hypertension, Self-monitoring, Blood Pressure, Telemonitoring,

For peer review only - http://bmjopen.bmj.com/site/about/guidelines.xhtml

Strengths and limitations of these trials:

- Based on current literature, these will be the largest randomised controlled trials of blood pressure self-monitoring in pregnancy completed to date.
- The pragmatic trial designs, with broad inclusion criteria, will make findings of both linked studies applicable to routine antenatal care.
- Linked process and health economic analysis will provide important contextual information regarding cost-effectiveness and acceptability.
- The trial is powered for the BUMP primary outcomes and some secondary outcomes but the effect of the intervention on rarer secondary maternal morbidity outcomes may still be uncertain.

For peer review only

Introduction

Raised blood pressure (BP) affects approximately 10% of pregnancies worldwide, almost half of these women develop pre-eclampsia.[1] Globally, around 15% of maternal mortality is due to pre-eclampsia so early detection and prevention are paramount.[2] In the UK, inadequate management of raised BP, in particular systolic hypertension, has previously been reported as a significant contributing factor to maternal deaths and although this has improved over the past decade, pre-eclampsia remains important. [3 4]

BP self-monitoring, where an individual measures their own BP outside of the clinical setting, allows for multiple measurements providing a better estimate of the underlying BP than intermittent clinic measurements. Such self-monitoring in pregnancy could improve both the detection and subsequent management of gestational hypertensive disorders including pre-eclampsia, whilst also empowering and engaging women in their own care.[5 6]

Women who are at higher risk for raised BP in pregnancy (e.g. due to age or previous medical history) may require more frequent monitoring.[7 8] BP can rise rapidly in pregnancy and hypertension may go undetected in between antenatal visits.[2] Once raised BP is detected during pregnancy, the clinical focus is on treating the hypertension, monitoring for development of pre-eclampsia and ensuring appropriate fetal surveillance.[7] Substantial resources are currently expended in monitoring such women, from the perspective of both the woman and the National Health Service (NHS).[9] If shown to be successful, self-monitoring could provide more accurate data for clinicians to use for treatment and management strategies, safely reduce the burden of multiple clinic visits for women, free up time for midwives, and therefore be a cost-effective option. Self-monitoring is easy to accomplish and is now commonplace in adults with hypertension outside of pregnancy. [10 11]

BP self-monitoring is therefore a potentially appealing option for both the detection and management of hypertension. We have previously shown feasibility and acceptability of self-monitoring of BP in pregnancy.[5 6] This trial aims to investigate self-monitoring in two groups by undertaking two linked, parallel group, randomised trials to evaluate whether self-monitoring of BP improves the detection of raised BP during higher risk pregnancies (BUMP 1) and whether self-monitoring improves BP control in women with hypertensive pregnancy (BUMP 2).

For peer review only - http://bmjopen.bmj.com/site/about/guidelines.xhtml

Methods

Study design and setting

The BUMP trials are two linked, multicentre, non-masked, parallel group, randomised controlled trials investigating self-monitoring of BP during pregnancy in the UK in secondary care maternity units (sites listed on https://clinicaltrials.gov/ct2/show/NCT03334149). The work is part of a larger programme of work investigating the use of self-monitoring and testing during pregnancy.

The BUMP 1 trial is a prospective non-masked randomised controlled trial of self-monitoring of BP in pregnancy for the detection of raised BP. Women at higher risk of pre-eclampsia will be recruited through antenatal clinics (*Table 1*). The consent process for BUMP 1 will include discussion of the transition to BUMP 2 should a woman develop raised BP.

The BUMP 2 trial is a prospective non-masked randomised controlled trial of self-monitoring of BP for the management of hypertension in pregnancy in women with chronic hypertension or gestational hypertension. Women may enter this study from BUMP 1 (maintaining original randomisation) or be recruited without prior involvement. See *Figure 1* for an overview of the study design.

There will be an initial external pilot phase including up to 50 women in order to test trial procedures prior to the commencement of full recruitment.

Intervention and controls groups

Usual care

Usual care will consist of pregnant women having their BP measured by their usual antenatal care team, and initiation and/or adjustment of medication (where appropriate) based on these measurements at the discretion of the health care professional.

Self-monitoring

Women randomised to self-monitoring will be provided with a monitor validated for use in pregnancy and pre-eclampsia (Microlife WatchBP Home) and instructions for its use. Participants will be enrolled on the telemonitoring system, a text/App based system and provided with clear instructions on its use. This system was developed from our pilot work with appropriate theoretical underpinnings and designed by the University of Oxford, Department of Engineering Science (R Band et al, paper submitted).

BUMP 1 participants will be asked to monitor their BP three times a week throughout their pregnancy. They will be instructed to sit quietly and comfortably for one minute, take two readings one minute apart and submit their second reading to the telemonitoring system. Participants will

For peer review only

receive a guideline with colour-coded instructions (*Figure 2*). If the second reading is outside the expected range, this will automatically trigger a request for a third reading (taken after 5 minutes) and persistent high or low readings will automatically trigger a message to ask the participant to contact their local maternity unit. If women have persistently high readings, they will be asked (via the telemonitoring system) to undertake daily readings for the rest of their pregnancy.

BUMP 2 participants will be asked to monitor their BP daily throughout their pregnancy. They will be instructed to take two measures (as above) and asked to submit their second reading to the telemonitoring system. Participants will receive a guideline with colour coded instructions (*Figure 3*). High or low second readings will automatically trigger a request for a third reading (taken after 5 minutes) and persistent high or low readings will automatically trigger a message for the participant to contact their local maternity unit.

On the basis of the results of our pilot work and recent systematic review, the same thresholds will be used for self-monitoring as clinic BP to trigger action (i.e. 140/90mmHg), therefore resulting in alerts to participating women via the telemonitoring system.[6 12]

To improve adherence to the intervention participants will receive weekly motivational messages, and these messages will be sent to women in the intervention group via the telemonitoring system (via the App or text message) based on the woman's preference. These are designed to provide support and education throughout the trial and were developed during our pilot work with patient and public involvement. Messages will be selected at random from a pool of messages (approved by the ethics committee). To monitor adherence and for safety the local clinical team will also receive warnings in real time via the telemonitoring system if a participant misses submitting readings, allowing them to approach the participant to troubleshoot any potential issues.

Participants will be provided with instructions in the use of the telemonitoring system at the baseline visit. Participants will send their readings to a centralised database, secured behind NHS firewalls, using a free SMS text message or app with web-based data entry back-up. Participants in BUMP 1 will receive a reminder after four days without submitting a reading and then one more reminder the following day if no further readings are entered. Participants in BUMP 2 will receive a reminder after two missed days of readings, with a further reminder the following day if no further readings are entered.

Each participant has the right to discontinue the intervention or withdraw from the trial at any time. In addition, an investigator may withdraw a participant from the trial at any time if the investigator considers it necessary for any reason including: ineligibility (either arising during the trial or retrospectively having been overlooked at screening), an adverse event which results in inability to continue to comply with trial procedures, withdrawal of consent and loss to follow up. If a woman

For peer review only - http://bmjopen.bmj.com/site/about/guidelines.xhtml

discontinues the intervention, withdraws or is withdrawn from the study at any point, her usual antenatal care will continue as all study procedures are additional rather than in place of usual care. Women who wish to discontinue the BP self-monitoring intervention will be asked if they are willing to participate in study follow-up. All data collected to the point of withdrawal will be retained in the study database. Unless a participant specifically withdraws consent, notes review will be conducted if possible, even where an individual has been lost to follow-up.

Study Outcomes

Outcome definitions

- The onset of hypertension/high BP will be defined as the earliest date recorded of sustained systolic BP ≥140 and/or diastolic BP ≥90 mm Hg from any community or hospital setting recorded in the notes review.
- Sustained is defined as at least two readings within 1 week (168 hours), with no minimum time between readings needing to be recorded.
- The 'earliest date recorded' will be for the second of these two readings.
- Additionally, a new prescription of antihypertensive medication for raised BP will be taken as a diagnosis of hypertension/high BP (in order to capture diagnoses where medication has been started but two raised BPs have not been recorded in the notes review for whatever reason). In this case the first date of prescription will be listed as the date of diagnosis.

Primary outcome for BUMP 1:

The primary outcome for BUMP 1 is the difference in time to a recording of raised BP by a health care professional between the usual care and self-monitoring groups.

Secondary outcomes for BUMP 1:

Secondary outcomes for BUMP 1 include analysis of the difference between usual care and self-monitoring groups as per *Table 3*:

Primary outcome for BUMP 2:

The primary outcome for BUMP 2 is the difference in mean systolic BPs, recorded by health care professionals from baseline to delivery, between usual care and self-monitoring groups.

For peer review only - http://bmjopen.bmj.com/site/about/guidelines.xhtml

Secondary Outcomes for BUMP 2:

Secondary outcomes for BUMP 2 include analysis of the difference between usual care and self-monitoring groups as per *Table 3*:

Participant Timeline

Members of the research team will provide a full verbal explanation and written description of the trial to women who meet the inclusion criteria (*Table 1 & 2*). The woman will be given sufficient time to consider the information, and to decide whether she will participate in the trial. Written informed consent will be sought from the woman and taken by an appropriately trained healthcare professional. Eligibility will be assessed and baseline data, including all demographics, maternal demographics and patient questionnaires (completed by the woman), will be entered on a web-based database by members of the research team. Participants will be randomised and instructed in the use of self-monitoring and provided with a BP monitor or instructed to follow usual care. Women randomised to usual care will not be prevented from self-monitoring although we will ask all women at final follow-up if they have self-monitored during the trial. Participants on both arms will follow their routine care clinic visits until delivery. Participants will be contacted at around 30 weeks' gestation (2 weeks after baseline if participants were recruited after 30 weeks) and asked either in person/over the phone/by post/ or via email to complete patient questionnaires. The remainder of antenatal care, in particular the timing and mode of delivery, will be left to the discretion of the responsible clinician. A medical notes review will be undertaken following the primary discharge for mother and baby. If a woman and/or her baby is still in hospital at 2 months from delivery or 2 months from her estimated date of delivery (whichever is longer), the admission time will be censored and data collected up to that point. Self-monitoring of BP will finish at admission in established labour or the end of pregnancy, whichever is sooner and participants will continue with standard postnatal care. Participants will be contacted at around 8 weeks post-delivery and asked either in person/over the phone/by post/ or via email to complete patient questionnaires and arrange for collection of the loaned BP monitor if not returned at delivery.   A schedule of participant enrolment, interventions and assessments in the trial is shown in *Table 4* and a flowchart of study visits is shown in *Figures 4 & 5*..

Sample size considerations

BUMP 1:

A sample size of 2262 (1131 per group) assuming a standard deviation (SD) of 40 days will allow detection of an effect size of 12 day's difference in time to detection of raised BP in pregnancy

For peer review only - http://bmjopen.bmj.com/site/about/guidelines.xhtml

between self-monitoring and control groups (the primary outcome of BUMP 1), with 90% power and 5% level of significance (2-sided) and assuming a 15% attrition rate. If the SD is 45 days, this sample size will allow detection of a difference of 14 days with more than 90% power and if the SD is 50 days then it will be sufficient to detect a difference of 16 days in time to detection of raised BP in pregnancy also with 90% power. The sample size was determined via a simulation method which modelled how many women would be expected to develop hypertension from a high risk group and how long time to detection would take in these women, determined from our pilot work in the BUMP study. Of the 2262 women recruited to BUMP 1, around 362 women are expected to develop hypertension. We will recruit a minimum of 2262 women to ensure adequate power.

BUMP 2:

A sample size of 256 per group will be sufficient to detect a 5mmHg difference in systolic BP between groups, accounting for 15% attrition and an SD of 16mmHg, based on data from the BUMP pilot and PELICAN studies, inflated from 14mmHg because BUMP 2 will include hypertensive women from BUMP 1 and those with chronic or gestational hypertension not previously randomised in BUMP 1.[6 16] The sample size was calculated using NCSS PASS v12.0.

Women randomised in BUMP 1 who develop hypertension will remain in their randomisation groups and move seamlessly into the BUMP 2 trial. This will occur automatically via the telemonitoring system and their inclusion in BUMP 2 assessed retrospectively. The precise numbers and proportion of women migrating from BUMP 1 to BUMP 2 will not be fixed. We will aim to recruit a minimum of 512 women directly into the BUMP 2 trial in addition to those that migrate in order to ensure adequate power: women who have chronic hypertension or have developed gestational hypertension and are not taking part in the BUMP 1 trial will be randomised to either usual care using clinic BP to guide treatment or BP self-monitoring with telemonitoring and automated feedback.

Both trials will be undertaken in approximately 15 maternity units in England to achieve the sample size in the anticipated time-frame. Our previous pilot trial has confirmed that women and clinicians are willing to participate in this type of randomised controlled trial and we have used recruitment estimates from this trial, together with trial management experience and expertise of the Co-Investigator Group to inform strategies to meet the required target size.[6]

Data management

An online clinical data management system will be used, with all clinical data entered directly onto the electronic CRFs and stored on a study specific database. Paper back-up forms will be available if the online database cannot be accessed. Data collected on paper forms will be entered on to the

For peer review only - http://bmjopen.bmj.com/site/about/guidelines.xhtml

database at the earliest opportunity, by single data entry. Patient-completed questionnaires will be completed directly on to the system by the participant wherever possible, with manual single data entry of paper copies if required. Telemonitored mHealth data is stored on a centralised database, secured behind NHS firewalls, designed and maintained by the Univeristy of Oxford, Department of Engineering Science.

Randomisation

Women will be randomised 1:1 to BP self-monitoring or usual care by the recruiting researcher. An independent statistician will generate a randomisation sequence for each trial, using permutated varying blocks and stratified by recruitment site and parity. Allocation of intervention will be carried out via the online data management system with telephone as back up.

Statistical analysis

General consideration for both trials:

The primary statistical analysis will be carried out on the basis of intention-to-treat (ITT). Thus, after randomisation participants will be analysed according to their allocated treatment group irrespective of what treatment they actually receive. We will endeavour to obtain full follow-up data on every participant to allow full ITT analysis, but we will inevitably experience the problem of missing data due to withdrawal, loss to follow up, or nonresponse questionnaire items. The results from the trial will be prepared as comparative summary statistics (e.g. difference in means) with 95% confidence intervals. The study results will be reported in accordance with the CONSORT 2010 statements and full detailed statistical analysis plans will be prepared before the final analysis by an independent statistician.

BUMP 1:

The primary analysis will determine whether there is a difference in the time to diagnosis between the randomised groups (usual care vs self-monitoring) and will be performed using a two-part model, such as the hurdle model.[17] The model takes into account participants who developed a raised BP or not and the time to raise BP, adjusting for stratification factors. This method has the advantage that all women who are recruited to BUMP 1 will contribute to the primary analysis. Continuous secondary outcomes, such as birth weight and length of stay, will be analysed by means of regression method adjusting for stratification factors. Binary secondary outcomes, such as the development of severe hypertension, development of any complications, and stillbirth rate, will be analysed by means of a log binomial model.

For peer review only - http://bmjopen.bmj.com/site/about/guidelines.xhtml

BUMP 2:

An ITT analysis will include women recruited to BUMP 1 who become hypertensive and women recruited de novo to BUMP 2 with GH and CH analysed separately. The primary analysis for BUMP 2 will in each case compare BPs between the intervention and control groups. Analysis of the BP outcome will be by means of a linear mixed effects model which can accommodate data where participants have repeated measurements, and also accounts for missing data (assuming data are missing at random (MAR)). Each of a participant's BP measures will be modelled by means of a linear mixed effects model, including random effects for participants and assuming an unstructured variance covariance matrix between measurements from the same participant. Time of BP measurement and randomised group, as well as their interaction term, will be included as fixed effects in the model. A fixed effect will also be included for whether the participant was from BUMP 1 or not in the GH analysis. As a sensitivity analysis inclusion from BUMP 1 or not will be tested as a moderator of the treatment effect. By means of appropriate contrasts, the difference in mean BP of participants in the randomised groups from both BUMP 1 and de novo recruitment will be compared.

BP load and area under the curve will be analysed as continuous outcomes by means of a linear regression model, adjusting for whether the participant originated from BUMP 1. Continuous secondary outcomes, such as birth weight and length of stay, will be analysed by means of regression model. Binary secondary outcomes, such as the development of severe hypertension, incidence of complications and incidence of stillbirth, will be analysed by means of a log binominal model.

Sub-Group Analyses:

For both BUMP 1 and BUMP 2, prespecified subgroups include eligibility for aspirin prescription to prevent pre-eclampsia (As per NICE); gestational age at baseline (≤20 weeks vs >20 weeks), parity (0 vs ≥1). In addition, a sensitivity analysis will compare those women with GH that are recruited de novo into BUMP 2 with those that migrate from BUMP 1.

Economic evaluation

The economic evaluation component aims to determine whether BP self-monitoring in addition to usual care represents value for money compared to usual care alone in a population of higher risk pregnant women (BUMP 1) and women with a diagnosis of hypertension during pregnancy (BUMP 2). Two separate within-trial cost-effectiveness analyses will be carried out alongside BUMP 1 and BUMP 2. In addition, a decision analytic model will be built to determine the long-term cost-

For peer review only - http://bmjopen.bmj.com/site/about/guidelines.xhtml

effectiveness of self-monitoring in pregnancy. A UK National Health Service (NHS) perspective will be adopted in all analyses.

The within-trial cost-utility analyses will use individual patient-level data with a time horizon up to two months following delivery. The main health outcome measure in the economic evaluation will be maternal quality-adjusted life years (QALYs) over the trial period. The calculation of a QALY profile for each woman will be informed using data from the health-related quality of life instrument EQ-5D-5L that will be collected at baseline, 30 weeks' gestation and eight weeks after birth.[13] A linear change between utility measures at each time period will be assumed when deriving an individual's QALY profile. Relevant healthcare resource use during pregnancy for mothers and their fetuses will include antenatal, intrapartum and postnatal care. Antenatal care utilisation will include primary and secondary care visits including hospital admissions. Intrapartum and related postnatal care before primary hospital discharge will include major procedures undertaken, length of stay and transfers for mothers and their babies. Primary and secondary care resource utilisation during the trial will be collected using case note review including routine and additional clinic visits to midwives and GPs. Unit costs will be extracted from primarily national sources.[18-20] The cost of using an automated monitor along with the telemonitoring system will be estimated and included in the cost analysis of the self-monitoring group in each trial. Mean maternal QALYs and costs (and associated uncertainty) will be estimated parametrically in the intervention and usual care group in each of the trials. If necessary, we will use multiple imputation methods to deal with missing EQ-5D-5L and resource use data over the trial period and will follow current guidance to conduct and report such analysis.[21]

The long-term decision analytical model will include a decision tree to represent women's pathways up to the first eight weeks post-delivery and a Markov model representing the history of disease of women with raised BP after that point, using annual cycles. The structure of the model will be based on models identified via a systematic review of previous economic evaluations of hypertensive patients in pregnancy (currently in progress with PROSPERO number: 123881). Observed risk factors, quality of life and health care resource utilisation for randomised participants in BUMP 1 and BUMP 2 will be used to inform the characteristics of a hypothetical cohort entering the model for each treatment pathway. Transition probabilities indicating movement across health states will be informed by cardiovascular risk predictions modified using the literature on risk following hypertensive pregnancy. Costs incurred annually in each health state will be obtained from the literature differentiating between acute and chronic disease health states. Health-related quality of life associated with each health state in the model will also be obtained from the literature.

For peer review only - http://bmjopen.bmj.com/site/about/guidelines.xhtml

Both the within-trial cost-effectiveness analysis and long-term model will be reported using current reporting standards for economic evaluations.[22] Mean differences in costs and QALYs between intervention and usual care arms in each trial and the model will be combined to generate an incremental cost-effectiveness ratio (ICER), presented as cost per QALY gained. Uncertainty around ICERs will be handled using confidence intervals (if appropriate) and cost-effectiveness acceptability curves. Uncertainty in the long-term model will be assessed using probabilistic sensitivity analysis. Recent guidance will also be used to report and interpret the results from the decision analytic model.[23]

Process Evaluation (qualitative and quantitative)

Alongside the trial we will undertake a process evaluation to assess both quantitative (including the outcomes listed in *Table 3* above) and qualitative (see below) aspects. Intervention fidelity will be assessed for women and participating professionals. This will include for the women: examining persistence with self-monitoring and comparing submitted readings and monitor downloads for a sub-set of women. For the professionals, a staff survey will assess practice in terms of utilisation of self-monitored BP readings during the trial.

In addition to the quantitative process evaluation, a qualitative study will be conducted within the trial to understand how BP self-monitoring in pregnancy is implemented and its acceptability in daily life and routine clinical practice. In-depth interviews will be completed with a sample of participating women in both BUMP 1 and BUMP 2 and healthcare professionals.[24]

Participating women: The study aims to include approximately 40 pregnant women across five sites, including a sample of up to eight women who decide not to take part or discontinue from the intervention and are willing to be interviewed.[25] The interviews with women will look to understand real-life implementation issues with BP self-monitoring. Women who have consented to contact at main trial enrolment will be contacted by phone/email/in person regarding the interviews and provided with a patient information sheet (PIS); they will be consented separately prior to the start of the interview. Additionally, an ethnographic study will follow a sample of 10-15 women who monitor their BP.[26] This ethnographic study will include longitudinal interviewing and non-participatory observations to understand in detail how women engage with home monitoring over time and whether and how home BP readings are incorporated into clinical encounters.[27 28]

Healthcare professionals: Interviews will be conducted with 30-40 midwives and obstetricians who are involved in BUMP 1 and BUMP 2.[25] The aim is to understand how self-monitoring is operationalised in practice and provide key information for the process evaluation.

For peer review only - http://bmjopen.bmj.com/site/about/guidelines.xhtml

Detailed field notes will be taken of observations. Interviews will be audio recorded so that content can be transcribed. Data collection and analysis will be guided by theory, including social cognitive theory, a "technology in practice" perspective and normalisation process theory as interpretative frameworks to understand how the trial intervention 'fits' within existing practices and systems, and how it becomes embedded (or not) in women's lives and healthcare professionals' routine work.[29 30 31 32]

Data Monitoring

Regular monitoring will be performed by the trial team according to Good Clinical Practice and Clinical Trial Unit standard operating procedures. Data will be evaluated for compliance with the protocol and accuracy in relation to source documents. Following written standard operating procedures, the monitors will verify that the clinical trial is conducted and data are generated, documented and reported in compliance with the protocol, GCP and the applicable regulatory requirements.

A Trial Steering Committee (TSC) and Data Monitoring Committee (DMC) will be convened and review trial progress every 6 months. The TSC will provide overall supervision of the trial and ensure its conduct is in accordance with the principles of GCP and the relevant regulations. The TSC will agree the trial protocol and provide advice to the investigators on all aspects of the trial. The TSC will include members who are independent of the investigators, in particular an independent chairperson. The independent DMC will inform the TSC regarding the accruing trial and safety data, to ensure trial site staff and participants are aware of any relevant safety information, and to advise the TSC regarding the appropriateness of continuation of the trial. Any consideration of the need for termination of the trial will be on the advice of the DMC and TSC who will consider the need for prospective criteria for termination.

Harms/ Potential Risks

During this randomised controlled trial participants will continue to receive "usual care" regardless of randomisation group, and thus we anticipate that the potential risks are low. Particular issues include the possibility of increased anxiety due to the study. Training of participants will cover repeated measurements in the case of unusually high or low readings. The participant guideline/booklet will give clear advice to women to contact the antenatal care team or other healthcare professional (e.g. General Practitioner (GP)) in the case of persistent high or low readings. The telemonitoring system will automatically provide this advice when high or low readings are sent

For peer review only - http://bmjopen.bmj.com/site/about/guidelines.xhtml

in. Women will continue to be seen by their clinical teams (midwives/ GPs/ obstetricians) at frequencies chosen by their clinicians throughout, regardless of randomisation group. Participants will be advised to seek immediate medical help if they experience any symptoms of pre-eclampsia, regardless of their BP readings.

It is not anticipated that the study intervention (self-monitoring of BP) should result in any adverse events (AEs) but collection of such events will be included in case such events occur so that they can be considered for causal links to the study. Only AEs that are clinically judged (by the supervising site Principal Investigator) as being caused by the trial intervention will be reported to the Clinical Trials Unit to raise appropriately to the sponsor/Research Ethics Committee that gave favourable opinion. In addition, any maternal death or stroke, fetal loss or neonatal death will be reported to the data monitoring committee regardless as to whether they are judged related. Side effects as stated in the British National Formulary will not be reported as AEs.

Confidentiality

The trial staff will ensure that the participants' anonymity is maintained in the trial database. The participants will be identified only by a participant identification number on all trial documents and any electronic database. Women's identifiers (names, address, phone number) will be held securely and separately from the Case Report Forms where they are needed to contact participants on an ongoing basis, e.g. for follow-up and return of BP monitor. Access to these data will be strictly on a need to know basis. All documents will be stored securely and only accessible by trial staff and authorised personnel. The study will comply with the Data Protection Act and General Data Protection Regulation (GDPR) 2018, which requires data to be anonymised as soon as it is practical to do so.

Patient and Public Involvement

Methods of patient approach, patient information sheet and consent form were all reviewed by patient representatives prior to formal approval. The relevant group for women with pre-eclampsia, "Action on Pre-Eclampsia" are formally represented through their Chief Executive Officer (MG) who is a co-investigator. The Trial Steering Committee includes an independent patient representative who is jointly responsible for overseeing the conduct of the trial with the rest of the committee.

For peer review only - http://bmjopen.bmj.com/site/about/guidelines.xhtml

Dissemination

All research outputs from this work will be published in peer-reviewed journals, presented at scientific conferences and lay and social media (e.g. Twitter, blogs). All trial data is the property of the Chief Investigator and will be stored until the end of the study, database lock and final analysis. All research data and documentation will be appropriately archived for at least five years. 'Patient friendly' study summary documents and infographics will be made available to all participants at the end of the trial via the study website.

DISCUSSION

This article describes the protocol for the BUMP trials. These randomised controlled trials will assess whether self-monitoring of BP during pregnancy, over and above usual care, can improve the detection of raised BP during pregnancy and whether self-monitoring can improve the control of BP in pregnancy hypertension.

Additional work will show whether self-monitoring BP is acceptable to pregnant women and their health care professionals and if it is cost-effective compared to usual care.

The results of the trial will provide data on the effects of self-monitoring and telemonitoring, over and above usual care, in pregnant women at risk for pre-eclampsia and women with hypertension. If self-monitoring of BP is found to be successful in the detection of hypertension in pregnancy and control of BP in pregnancy hypertension, then it would be applicable to a large group of pregnant women in the UK but also worldwide.

Current trial status

The trials commenced recruitment on 11th September 2017 (external pilot) with main recruitment starting on (22nd November 2017) and recruitment is planned to continue until end of September 2019. Data collection will continue to mid 2020.

ABBREVIATIONS

BP: Blood Pressure; EQ-5D-5L: European Questionnaire 5 Dimensions; GP: General Practitioner; mmHg: Millimetre of mercury; NHS: National Health Service; NICE: National Institute for Health and Care Excellence; PC-CTU: Primary Care – Clinical Trials Unit

For peer review only - http://bmjopen.bmj.com/site/about/guidelines.xhtml

DECLARATIONS

Ethics approval and consent to participate

The protocol, informed consent form, participant information sheet and all other participant-facing material have been approved by the Research Ethics Committee (West Midlands - South Birmingham: ref 17/WM/0241), host institution(s) and Health Research Authority. Any subsequent protocol amendments will be agreed with both sponsor and ethics committee prior to implementation. The study sponsor reviewed and ensured all indemnity and insurance requirements for the trial were in place prior to the start of recruitment which would operate in the event of any participant suffering harm as a result of their involvement in the research. NHS indemnity operates in respect of the clinical treatment which is provided. Participants will provide written informed consent prior to enrolment. An independent trial steering group will monitor study progress assisted by an independent data monitoring committee and will periodically review the study.

Competing interests

RJM has previously received BP monitors from Omron for research purposes. The BP monitors for the current trials were purchased from the manufacturer (Microlife) at commercial prices. The authors declare no conflicts of interest.

Funding

This work is funded from a National Institute for Health Research (NIHR) Programme grant for applied research (RP-PG-1209-10051) and an NIHR Professorship awarded to RJM (NIHR-RP-R2-12-015). RJM and KT receives funding from the National Institute for Health Research (NIHR) Collaboration for Leadership in Applied Health Research and Care Oxford at Oxford Health NHS Foundation Trust. JS is a National Institute for Health Research (NIHR) Senior Investigator and supported by the National Institute for Health Research (NIHR) Collaboration for Leadership in Applied Health Research and Care South London (NIHR CLAHRC South London) at King's College Hospital NHS Foundation Trust. Service support costs will be administered through the NIHR Clinical Research Network. The views expressed in this publication are those of the authors and not necessarily those of the NHS, the NIHR or the Department of Health and social care.

Authors' contributions

RJM had the original idea and applied for funding with KT, LC, LMY, LH, ORA, CC, MG, SG, JH, PL, CM, LM, JS, LT and LY. MF, GD, RJM, LC and KT wrote the first draft. AN and LMY provided the sample size calculations and statistical analysis section. LH provided the qualitative section. ORA and LA provided

For peer review only - http://bmjopen.bmj.com/site/about/guidelines.xhtml

the economic evaluation section. GD is the trial manager (previously MF). All authors subsequently critically edited the manuscript. RJM will be guarantor for the manuscript. All authors have read and approved the final manuscript.

Acknowledgements

The authors acknowledge the support of the Primary Care Clinical Trials Unit; Margaret Glogowska, Jacqui Williams and Tricia Carver have served as patient representatives for the trial management and steering groups. Additional members of the trial steering group are Laura Magee (chair), Jim Thornton, John Norrie and Tim Coleman. Members of the data monitoring committee are Nigel Simpson (chair), Julia Sanders and Miliça Bucknall. The sponsor and funder had no role in the study design, writing of the paper; or the decision to submit this protocol for publication, which was made jointly by the authors who have all approved the final manuscript.

Protocol Version: V2.0 26.11.2018

Trial Sponsor: University of Oxford

Contact name: Ms Heather House

Address: Clinical Trials and Research Governance, Joint Research Office, Block 60, Churchill Hospital, University of Oxford, Oxford, OX3 7LE

Email: ctrg@admin.ox.ac.uk

For peer review only

FIGURES AND TABLES

Table 1: BUMP 1 Inclusion and Exclusion Criteria

**Inclusion Criteria:**
• Participant is willing and able to give informed consent for participation in the trial
• Pregnant woman, aged 18 years or above between 16^{+0} to 24^{+0} weeks
• Able and willing to comply with trial requirements
• Willing to allow her GP and consultant, if appropriate, to be notified of participation in the trial
• At higher risk for hypertension in pregnancy / pre-eclampsia defined as one or more of the following risk factors:<br>○ Age 40 years or older<br>○ Nulliparity<br>○ Pregnancy interval of more than 10 years<br>○ Family history of pre-eclampsia<br>○ Previous history of pre-eclampsia or gestational hypertension<br>○ Body mass index 30 kg/m2 or above at booking<br>○ Chronic kidney disease<br>○ Twin pregnancy<br>○ Pre-pregnancy diabetes (Type 1 or 2)<br>○ Autoimmune Disease (e.g. systemic lupus erythematosus or antiphospholipid disease)
**Exclusion Criteria:**
• Chronic Hypertension

For peer review only

Table 2: BUMP 2 Inclusion and Exclusion Criteria

**Inclusion Criteria:**
• Women with chronic hypertension (defined as sustained systolic BP≥140 mmHg and/or diastolic BP≥90 mmHg, present at booking or before 20 weeks gestation, or receiving antihypertensive treatment outside pregnancy and/or at time of referral), recruited up to 37^{+0} weeks' gestation.<br>*OR*<br>• Women with gestational hypertension after 20 weeks gestation (defined as sustained systolic BP≥140 mmHg and/or diastolic BP≥90 mmHg), recruited at 20^{+0} to 37^{+0} weeks' gestation.<br>*AND*
• Participant is willing and able to give informed consent for participation in the trial.
• Woman aged 18 years or above
• Willing to allow her GP and consultant, if appropriate, to be notified of participation
**Exclusion Criteria:**
• Anticipated inpatient admission considered likely to lead to imminent delivery (within the next 48 hours)

For peer review only - http://bmjopen.bmj.com/site/about/guidelines.xhtml

Table 3: Secondary Outcomes for BUMP 1 and BUMP 2

**Maternal Outcomes**
• Severe hypertension (systolic BP ≥160mmHg and/or diastolic BP ≥110mmHg)
• Pre-eclampsia or Gestational Hypertension
• Serious maternal complications including eclampsia, transient ischemic attack or stroke, pulmonary oedema, renal failure, blood transfusion, HELLP syndrome, Liver Involvement, Haematological Involvement and death
• Onset of labour
• Change in maternal quality of life from randomisation (measured using the EQ-5D-5L questionnaire)[13]
• Diastolic blood pressure*
• Area under the blood pressure curve*
• Proportion of systolic readings above 140mmHg*
**Perinatal Outcomes**
• Stillbirths
• Early neonatal deaths
• Gestation at delivery
• Mode of delivery
• Birth weight of the baby, including centile
• Small for gestational age infants (<10th and <3rd centile)
• Neonatal unit admissions, including length of stay
**Process Outcomes**
• Change in health behaviours (questionnaire)
• Fidelity to monitoring schedule
• Change in State Trait Anxiety Inventory short form (STAI-6) anxiety questionnaire[14]
• Health service costs
• Cost per maternal quality-adjusted life year gained over trial period
• Change in adherence to medication (MARS questionnaire)*[15]
• Qualitative data gathered from participating women and healthcare professionals

Outcomes common to both trials apart from *only Assessed on BUMP 2 Participants

For peer review only - http://bmjopen.bmj.com/site/about/guidelines.xhtml

Table 4: Schedule of interventions and assessments.

	STUDY PERIOD (16 weeks to 8 weeks post-delivery)			
**Study Visit**	**Trial Entry/Baseline**	**Antenatal Follow Up**	**Notes Review**	**Postnatal Follow Up**
**TIMEPOINT**	***20±4 Weeks' Gestation***	**30±3 Weeks' *Gestation***	***40+4 Weeks' (Post Delivery)***	***40+8 Weeks' (Post Delivery)***
**Informed Consent**	X			
**Eligibility Assessment**	X			
**Demographics; Including age, race, education**	X			
**Maternal Demographics; including gestation, EDD and Parity**	X			
**Patient Questionnaires; Including EQ-5D-5L and STAI**	X	X		X
**Randomisation**	X			
**Recording Home Blood Pressure**	Self-Monitoring group will record and submit reading between baseline and delivery			
**Recording Clinic Blood Pressures**			X	
**Maternal and Perinatal Outcomes**			X	
**Secondary Outcome Data**			X	
**Return of BP Monitor**			BP Monitors will be returned at/or post delivery	

For peer review only

References

1. Khan KS, Wojdyla D, Say L, et al. WHO analysis of causes of maternal death: a systematic review. *Lancet* 2006;367(9516):1066-74. doi: 10.1016/S0140-6736(06)68397-9 [published Online First: 2006/04/04]
2. Douglas KA, Redman CW. Eclampsia in the United Kingdom. *BMJ* 1994;309(6966):1395-400. [published Online First: 1994/11/26]
3. Cantwell R, Clutton-Brock T, Cooper G, et al. Saving Mothers' Lives: Reviewing maternal deaths to make motherhood safer: 2006-2008. The Eighth Report of the Confidential Enquiries into Maternal Deaths in the United Kingdom. *BJOG* 2011;118 Suppl 1:1-203. doi: 10.1111/j.1471-0528.2010.02847.x [published Online First: 2011/03/05]
4. Knight M, Tuffnell, D, Kenyon, S, Shakespeare, J., Gray, R., & Kurinczuk, J. J. Saving lives, improving mothers' care: Surveillance of maternal deaths in the UK 2011-13 and lessons learned to inform maternity care from the UK and Ireland. Confidential Enquiries into Maternal Deaths and Morbidity 2009-13. *National Perinatal Epidemiology Unit, University of Oxford* 2015
5. Hinton L, Tucker KL, Greenfield SM, et al. Blood pressure self-monitoring in pregnancy (BuMP) feasibility study; a qualitative analysis of women's experiences of self-monitoring. *BMC Pregnancy Childbirth* 2017;17(1):427. doi: 10.1186/s12884-017-1592-1 [published Online First: 2017/12/21]
6. Tucker KL, Taylor KS, Crawford C, et al. Blood pressure self-monitoring in pregnancy: examining feasibility in a prospective cohort study. *BMC Pregnancy Childbirth* 2017;17(1):442. doi: 10.1186/s12884-017-1605-0 [published Online First: 2017/12/30]
7. Hypertension in pregnancy: diagnosis and management (NICE Guideline 107): National Institute for Health and Care Excellence; 2010 [Available from: https://www.nice.org.uk/guidance/cg107 accessed 20 November 2018.
8. Antenatal care for uncomplicated pregnancies (NICE Guideline 62): National Institute for Health and Care Excellence; 2008 [Available from: https://www.nice.org.uk/guidance/cg62 accessed 20 November 2018.
9. Maternity Services in England: The National Audit Office. The Stationery Office, Department of Health. London., 2013.
10. Baral-Grant S, Haque MS, Nouwen A, et al. Self-Monitoring of Blood Pressure in Hypertension: A UK Primary Care Survey. *Int J Hypertens* 2012;2012:582068. doi: 10.1155/2012/582068 [published Online First: 2011/10/21]
11. Bray EP, Holder R, Mant J, et al. Does self-monitoring reduce blood pressure? Meta-analysis with meta-regression of randomized controlled trials. *Ann Med* 2010;42(5):371-86. doi: 10.3109/07853890.2010.489567 [published Online First: 2010/05/28]
12. Tucker KL, Bankhead C, Hodgkinson J, et al. How Do Home and Clinic Blood Pressure Readings Compare in Pregnancy? *Hypertension* 2018;72(3):686-94. doi: 10.1161/HYPERTENSIONAHA.118.10917 [published Online First: 2018/10/26]
13. Herdman M, Gudex C, Lloyd A, et al. Development and preliminary testing of the new five-level version of EQ-5D (EQ-5D-5L). *Qual Life Res* 2011;20(10):1727-36. doi: 10.1007/s11136-011-9903-x [published Online First: 2011/04/12]
14. Marteau TM, Bekker H. The development of a six-item short-form of the state scale of the Spielberger State—Trait Anxiety Inventory (STAI). 1992;31(3):301-06. doi: 10.1111/j.2044-8260.1992.tb00997.x
15. Horne R, Weinman J. Patients' beliefs about prescribed medicines and their role in adherence to treatment in chronic physical illness. *J Psychosom Res* 1999;47(6):555-67. doi: Doi 10.1016/S0022-3999(99)00057-4
16. Chappell LC, Duckworth S, Seed PT, et al. Diagnostic Accuracy of Placental Growth Factor in Women With Suspected Preeclampsia A Prospective Multicenter Study. *Circulation* 2013;128(19):2121-31. doi: 10.1161/Circulationaha.113.003215

For peer review only - http://bmjopen.bmj.com/site/about/guidelines.xhtml

17. Cragg JG. Some Statistical Models for Limited Dependent Variables with Application to the Demand for Durable Goods. *Econometrica* 1971;39(5):829-44.
18. British National Formulary 2017 [Available from: Available at https://bnf.nice.org.uk/ accessed (Last accessed: 22 January 2019).
19. Curtis LaB, A. Unit Costs of Health and Social Care. University of Kent, Personal Social Services Research Unit. 2017 [Available from: Available at https://www.pssru.ac.uk/project-pages/unit-costs/unit-costs-2017/ accessed (Last accessed: 17 January 2019).
20. NHS reference cost 2017-18 [Available from: Available at https://improvement.nhs.uk/resources/reference-costs/#rc1718 accessed (Last accessed: 17 January 2019).
21. Faria R, Gomes M, Epstein D, et al. A guide to handling missing data in cost-effectiveness analysis conducted within randomised controlled trials. *Pharmacoeconomics* 2014;32(12):1157-70. doi: 10.1007/s40273-014-0193-3 [published Online First: 2014/07/30]
22. Husereau D, Drummond M, Petrou S, et al. Consolidated Health Economic Evaluation Reporting Standards (CHEERS)--explanation and elaboration: a report of the ISPOR Health Economic Evaluation Publication Guidelines Good Reporting Practices Task Force. *Value Health* 2013;16(2):231-50. doi: 10.1016/j.jval.2013.02.002 [published Online First: 2013/03/30]
23. Caro JJ, Briggs AH, Siebert U, et al. Modeling good research practices--overview: a report of the ISPOR-SMDM Modeling Good Research Practices Task Force--1. *Value Health* 2012;15(6):796-803. doi: 10.1016/j.jval.2012.06.012 [published Online First: 2012/09/25]
24. Jamshed S. Qualitative research method-interviewing and observation. *J Basic Clin Pharm* 2014;5(4):87-8. doi: 10.4103/0976-0105.141942 [published Online First: 2014/10/16]
25. Sandelowski M. Sample size in qualitative research. *Res Nurs Health* 1995;18(2):179-83. [published Online First: 1995/04/01]
26. Reeves S, Kuper A, Hodges BD. Qualitative research methodologies: ethnography. *BMJ* 2008;337:a1020. doi: 10.1136/bmj.a1020 [published Online First: 2008/08/09]
27. Hermanowicz JC. The Longitudinal Qualitative Interview. *Qual Sociol* 2013;36(2):189-208. doi: 10.1007/s11133-013-9247-7
28. Williams J. Nonparticipant observation 2008:561-62.
29. Timmermans S, Berg M. The practice of medical technology. *Sociol Health Illn* 2003; 25:97-114. [published Online First: 2003/09/23]
30. Yardley L, Morrison L, Bradbury K, et al. The person-based approach to intervention development: application to digital health-related behavior change interventions. *J Med Internet Res* 2015;17(1):e30. doi: 10.2196/jmir.4055 [published Online First: 2015/02/03]
31. May CR, Mair FS, Dowrick CF, et al. Process evaluation for complex interventions in primary care: understanding trials using the normalization process model. *BMC Fam Pract* 2007; 8:42. doi: 10.1186/1471-2296-8-42 [published Online First: 2007/07/26]
32. F. S. Understanding and Using Health Experiences: improving patient care: Observing interactions as an approach to understanding patients' experiences: Oxford Oxford University Press 2013.

For peer review only - http://bmjopen.bmj.com/site/about/guidelines.xhtml

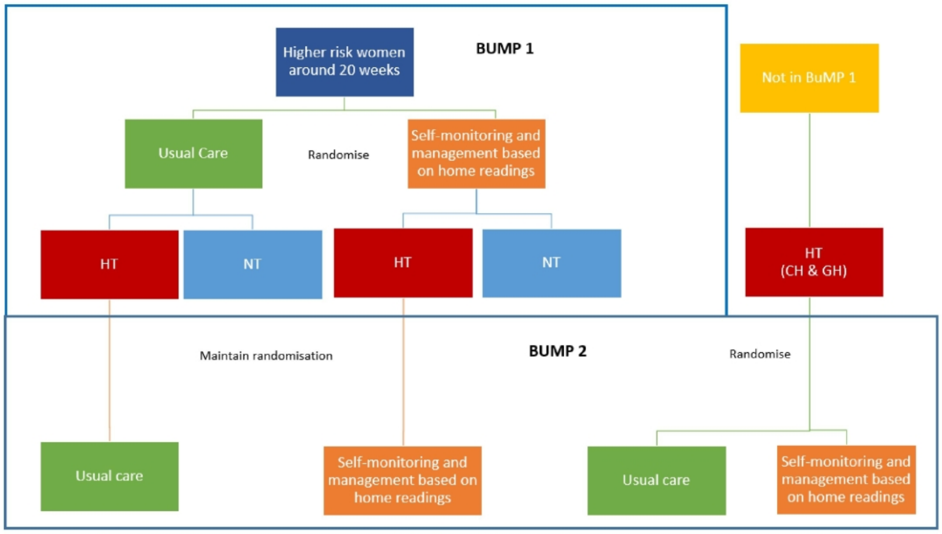

Figure 1: Study Design
HT – Hypertension, NT – Normotension, CH - Chronic Hypertension, GH – Gestational Hypertension

274x162mm (300 x 300 DPI)

LEVEL	BLOOD PRESSURE /mmHg	ACTION
HIGH	SYS 150 or more<br>OR<br>DIA 100 or more	Your blood pressure is high<br><br>Sit quietly for 5 minutes then measure it again and send in the reading.<br><br>Contact your maternity unit for urgent assessment today (within 4 hours) and continue to monitor your BP daily.
RAISED	SYS 140-149<br>OR<br>DIA 90-99	Your blood pressure is raised<br><br>Sit quietly for 5 minutes then measure it again and send in the reading.<br><br>If your repeated reading is raised please contact your maternity unit within 24 hours and continue to monitor your BP daily.
HIGH NORMAL	SYS 135-139<br>OR<br>DIA 85-89	Your blood pressure is normal but moving towards the raised threshold<br><br>Sit quietly for 5 minutes then measure it again and send in the reading.<br><br>If your repeat reading is still high-normal, please monitor your blood pressure daily.
NORMAL	SYS 110-134<br>OR<br>DIA 70-84	Your blood pressure is normal.<br><br>Continue blood pressure monitoring and your current care
LOW	SYS 109 or less<br>AND<br>DIA 69 or less	Your blood pressure is low. Repeat once more in 5 minutes.<br><br>If you are taking blood pressure medication, contact your maternity unit within 24 hours or within 4 hours if you feel unwell (e.g. dizzy or faint).<br><br>If you are not taking medication and you are feeling well this blood pressure does not need any further action.

Figure 2: Blood Pressure Interpretation Chart for BUMP 1

165x207mm (300 x 300 DPI)

LEVEL	BLOOD PRESSURE /mmHg	ACTION
**HIGH**	SYS 150 or more<br>OR<br>DIA 100 or more	Your blood pressure is high<br>Sit quietly for 5 minutes then measure it again and send in the reading.<br>Contact your maternity unit for urgent assessment today (within 4 hours) and continue to monitor your BP daily.
**RAISED**	SYS 140-149<br>OR<br>DIA 90-99	Your blood pressure is raised<br>Sit quietly for 5 minutes then measure it again and send in the reading.<br>If your repeated reading is raised please contact your maternity unit within 24 hours and continue to monitor your BP daily.
**NORMAL**	SYS 110-139<br>OR<br>DIA 70-89	Your blood pressure is normal.<br>Continue blood pressure monitoring and your current care
**LOW**	SYS 109 or less<br>AND<br>DIA 69 or less	Your blood pressure is low. Repeat once more in 5 minutes.<br>If you are taking blood pressure medication, contact your maternity unit within 24 hours or within 4 hours if you feel unwell (e.g. dizzy or faint).<br>If you are not taking medication and you are feeling well this blood pressure does not need any further action.

Figure 3: Blood Pressure Interpretation Chart for BUMP 2

171x162mm (300 x 300 DPI)

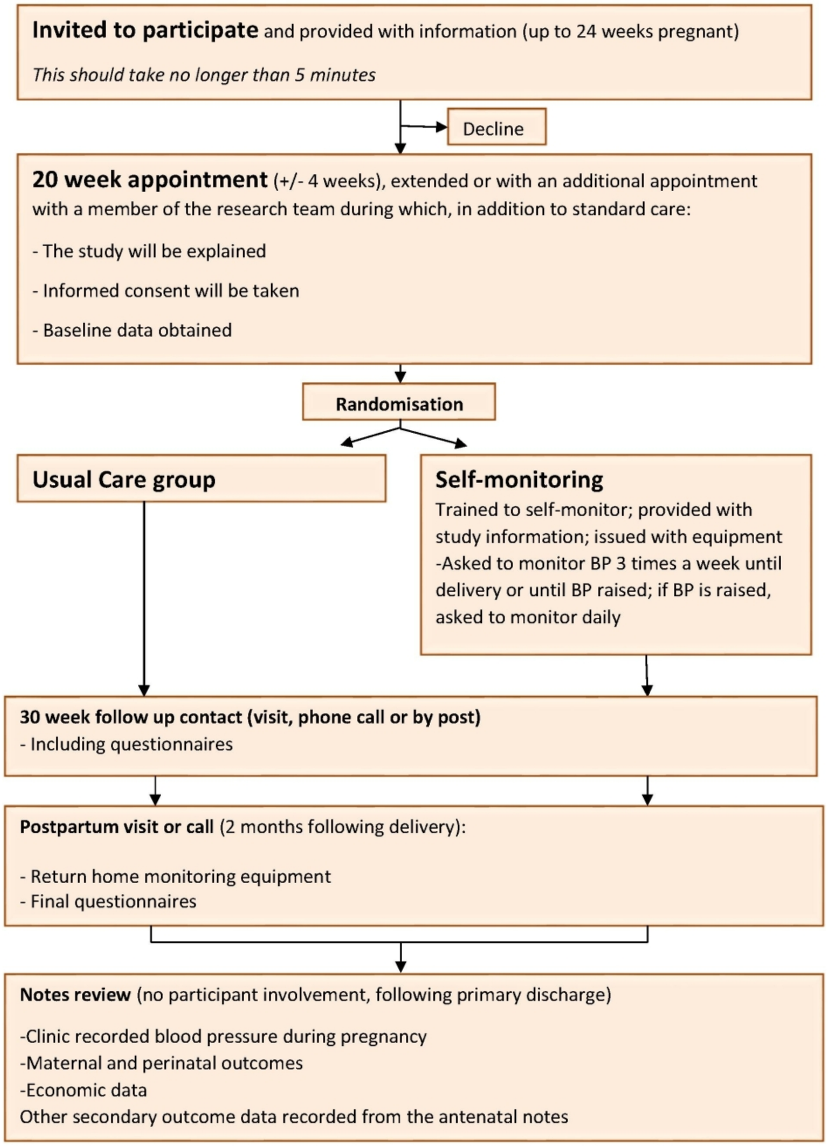

Figure 4: BUMP 1 Study Visit Chart

169x228mm (300 x 300 DPI)

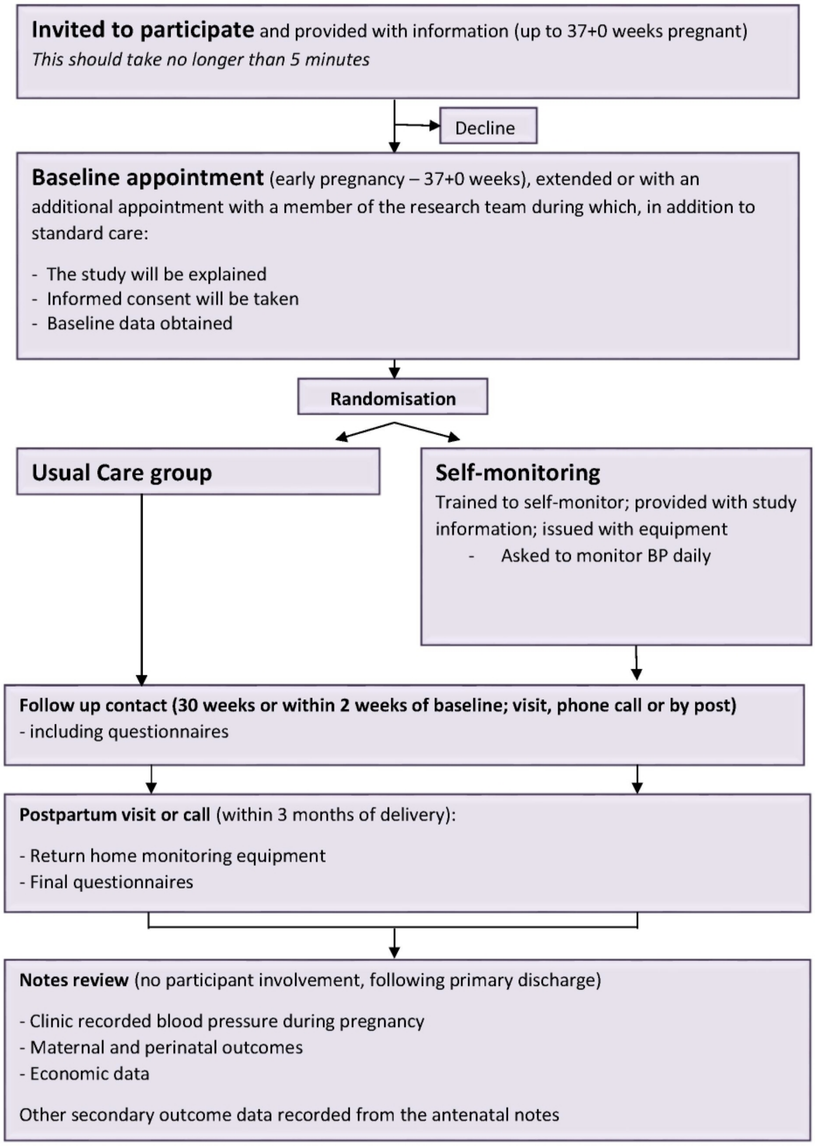

Figure 5: BUMP 2 Study Visit Chart

168x232mm (300 x 300 DPI)

SPIRIT

STANDARD PROTOCOL ITEMS: RECOMMENDATIONS FOR INTERVENTIONAL TRIALS

SPIRIT 2013 Checklist: Recommended items to address in a clinical trial protocol and related documents*

Section/item	ItemNo	Description	Addressed on page number
**Administrative information**			
Title	1	Descriptive title identifying the study design, population, interventions, and, if applicable, trial acronym	1
Trial registration	2a	Trial identifier and registry name. If not yet registered, name of intended registry	2, 5
	2b	All items from the World Health Organization Trial Registration Data Set	NA
Protocol version	3	Date and version identifier	20
Funding	4	Sources and types of financial, material, and other support	19, 20
Roles and responsibilities	5a	Names, affiliations, and roles of protocol contributors	1, 20
	5b	Name and contact information for the trial sponsor	20
	5c	Role of study sponsor and funders, if any, in study design; collection, management, analysis, and interpretation of data; writing of the report; and the decision to submit the report for publication, including whether they will have ultimate authority over any of these activities	19, 20

For peer review only

	5d	Composition, roles, and responsibilities of the coordinating centre, steering committee, endpoint adjudication committee, data management team, and other individuals or groups overseeing the trial, if applicable (see Item 21a for data monitoring committee)	16, 19
**Introduction**			
Background and rationale	6a	Description of research question and justification for undertaking the trial, including summary of relevant studies (published and unpublished) examining benefits and harms for each intervention	4
	6b	Explanation for choice of comparators	4, 6 - 8
Objectives	7	Specific objectives or hypotheses	4
Trial design	8	Description of trial design including type of trial (eg, parallel group, crossover, factorial, single group), allocation ratio, and framework (eg, superiority, equivalence, noninferiority, exploratory)	5
**Methods: Participants, interventions, and outcomes**			
Study setting	9	Description of study settings (eg, community clinic, academic hospital) and list of countries where data will be collected. Reference to where list of study sites can be obtained	5
Eligibility criteria	10	Inclusion and exclusion criteria for participants. If applicable, eligibility criteria for study centres and individuals who will perform the interventions (eg, surgeons, psychotherapists)	5, 6
Interventions	11a	Interventions for each group with sufficient detail to allow replication, including how and when they will be administered	6 - 8

For peer review only

For peer review only - http://bmjopen.bmj.com/site/about/guidelines.xhtml

	11b	Criteria for discontinuing or modifying allocated interventions for a given trial participant (eg, drug dose change in response to harms, participant request, or improving/worsening disease)	8
	11c	Strategies to improve adherence to intervention protocols, and any procedures for monitoring adherence (eg, drug tablet return, laboratory tests)	7
	11d	Relevant concomitant care and interventions that are permitted or prohibited during the trial	10
Outcomes	12	Primary, secondary, and other outcomes, including the specific measurement variable (eg, systolic blood pressure), analysis metric (eg, change from baseline, final value, time to event), method of aggregation (eg, median, proportion), and time point for each outcome. Explanation of the clinical relevance of chosen efficacy and harm outcomes is strongly recommended	8, 9
Participant timeline	13	Time schedule of enrolment, interventions (including any run-ins and washouts), assessments, and visits for participants. A schematic diagram is highly recommended (see Figure)	10, 11
Sample size	14	Estimated number of participants needed to achieve study objectives and how it was determined, including clinical and statistical assumptions supporting any sample size calculations	11, 12
Recruitment	15	Strategies for achieving adequate participant enrolment to reach target sample size	12

For peer review only - http://bmjopen.bmj.com/site/about/guidelines.xhtml

Methods: Assignment of interventions (for controlled trials)

Allocation:

Sequence generation	16a	Method of generating the allocation sequence (eg, computer-generated random numbers), and list of any factors for stratification. To reduce predictability of a random sequence, details of any planned restriction (eg, blocking) should be provided in a separate document that is unavailable to those who enrol participants or assign interventions	12
Allocation concealment mechanism	16b	Mechanism of implementing the allocation sequence (eg, central telephone; sequentially numbered, opaque, sealed envelopes), describing any steps to conceal the sequence until interventions are assigned	12
Implementation	16c	Who will generate the allocation sequence, who will enrol participants, and who will assign participants to interventions	12
Blinding (masking)	17a	Who will be blinded after assignment to interventions (eg, trial participants, care providers, outcome assessors, data analysts), and how	NA
	17b	If blinded, circumstances under which unblinding is permissible, and procedure for revealing a participant's allocated intervention during the trial	NA

Methods: Data collection, management, and analysis

Data collection methods	18a	Plans for assessment and collection of outcome, baseline, and other trial data, including any related processes to promote data quality (eg, duplicate measurements, training of assessors) and a description of study instruments (eg, questionnaires, laboratory tests) along with their reliability and validity, if known. Reference to where data collection forms can be found, if not in the protocol	10, 12

For peer review only - http://bmjopen.bmj.com/site/about/guidelines.xhtml

	18b	Plans to promote participant retention and complete follow-up, including list of any outcome data to be collected for participants who discontinue or deviate from intervention protocols	7, 8
Data management	19	Plans for data entry, coding, security, and storage, including any related processes to promote data quality (eg, double data entry; range checks for data values). Reference to where details of data management procedures can be found, if not in the protocol	12
Statistical methods	20a	Statistical methods for analysing primary and secondary outcomes. Reference to where other details of the statistical analysis plan can be found, if not in the protocol	13, 14
	20b	Methods for any additional analyses (eg, subgroup and adjusted analyses)	14
	20c	Definition of analysis population relating to protocol non-adherence (eg, as randomised analysis), and any statistical methods to handle missing data (eg, multiple imputation)	13
**Methods: Monitoring**			
Data monitoring	21a	Composition of data monitoring committee (DMC); summary of its role and reporting structure; statement of whether it is independent from the sponsor and competing interests; and reference to where further details about its charter can be found, if not in the protocol. Alternatively, an explanation of why a DMC is not needed	17
	21b	Description of any interim analyses and stopping guidelines, including who will have access to these interim results and make the final decision to terminate the trial	17

Harms	22	Plans for collecting, assessing, reporting, and managing solicited and spontaneously reported adverse events and other unintended effects of trial interventions or trial conduct	17
Auditing	23	Frequency and procedures for auditing trial conduct, if any, and whether the process will be independent from investigators and the sponsor	16
**Ethics and dissemination**			
Research ethics approval	24	Plans for seeking research ethics committee/institutional review board (REC/IRB) approval	19
Protocol amendments	25	Plans for communicating important protocol modifications (eg, changes to eligibility criteria, outcomes, analyses) to relevant parties (eg, investigators, REC/IRBs, trial participants, trial registries, journals, regulators)	19
Consent or assent	26a	Who will obtain informed consent or assent from potential trial participants or authorised surrogates, and how (see Item 32)	10
	26b	Additional consent provisions for collection and use of participant data and biological specimens in ancillary studies, if applicable	15, 16
Confidentiality	27	How personal information about potential and enrolled participants will be collected, shared, and maintained in order to protect confidentiality before, during, and after the trial	18
Declaration of interests	28	Financial and other competing interests for principal investigators for the overall trial and each study site	19, 20
Access to data	29	Statement of who will have access to the final trial dataset, and disclosure of contractual agreements that limit such access for investigators	18

For peer review only - http://bmjopen.bmj.com/site/about/guidelines.xhtml

Ancillary and post-trial care	30	Provisions, if any, for ancillary and post-trial care, and for compensation to those who suffer harm from trial participation	10, 19
Dissemination policy	31a	Plans for investigators and sponsor to communicate trial results to participants, healthcare professionals, the public, and other relevant groups (eg, via publication, reporting in results databases, or other data sharing arrangements), including any publication restrictions	18
	31b	Authorship eligibility guidelines and any intended use of professional writers	20
	31c	Plans, if any, for granting public access to the full protocol, participant-level dataset, and statistical code	NA
**Appendices**			
Informed consent materials	32	Model consent form and other related documentation given to participants and authorised surrogates	NA
Biological specimens	33	Plans for collection, laboratory evaluation, and storage of biological specimens for genetic or molecular analysis in the current trial and for future use in ancillary studies, if applicable	NA

*It is strongly recommended that this checklist be read in conjunction with the SPIRIT 2013 Explanation & Elaboration for important clarification on the items. Amendments to the protocol should be tracked and dated. The SPIRIT checklist is copyrighted by the SPIRIT Group under the Creative Commons "Attribution-NonCommercial-NoDerivs 3.0 Unported" license.

For peer review only

BMJ Open

Blood pressure monitoring in high risk pregnancy to improve the detection and monitoring of hypertension (The BUMP 1&2 trials): protocol for two linked randomised controlled trials.

Journal:	*BMJ Open*
Manuscript ID	bmjopen-2019-034593.R1
Article Type:	Protocol
Date Submitted by the Author:	20-Dec-2019

Complete List of Authors:	Dougall, Greig; University of Oxford, Nuffield department of Primary Care Health Sciences<br>Franssen, Marloes; University of Oxford, Nuffield department of Primary Care Health Sciences<br>Tucker, Katherine; University of Oxford, Nuffield Department of Primary Care<br>Yu, Ly-Mee; University of Oxford, Department of Primary Care Health Sciences<br>Hinton, Lisa; University of Oxford, Health Experiences Research Group, Nuffield Department of Primary Care Health Sciencs<br>Rivero-Arias, Oliver; University of Oxford, Nuffield Department of Population Health<br>Abel, Lucy; University of Oxford<br>Allen, Julie; University of Oxford, Nuffield Department of Primary Care Health Sciences<br>Band, Rebecca; University of Southampton, Psychology<br>Chisholm, Alison; University of Oxford Nuffield Department of Population Health, Maternal Health and Care<br>Crawford, Carole; University of Oxford, Nuffield department of Primary Care Health Sciences<br>Green, Marcus; Action on Pre-eclampsia<br>Greenfield, Sheila; University of Birmingham, Primary Care Clinical Sciences<br>Hodgkinson, James ; University of Birmingham, Primary Care Clinical Sciences, Institute of Applied Health Research<br>Leeson, Paul; University of Oxford, Division of Cardiovascular Medicine<br>McCourt, Christine; Department of Midwifery and Child health, City University London<br>MacKillop, Lucy; Oxford University Hospitals NHS Trust, Nuffield Department of Obstetrics & Gynaecology<br>Nickless, Alecia; University of Oxford, Nuffield Department of Primary Care Health Sciences<br>Sandall, Jane; Kings College, London, Health and Social Care Research Division<br>Santos, Mauro; University of Oxford, Institute of Biomedical Engineering, Department of Engineering Science<br>Tarassenko, Lionel; University of Pittsburgh, Engineering Science<br>Velardo, Carmelo; University of Oxford, Institute of Biomedical

	Engineering<br>Wilson, Hannah; King’s College London, Women’s Health Academic Centre<br>Yardley, Lucy; University of Southampton, Psychology; University of Bristol, School of Health Sciences<br>Chappell, Dr Lucy; King’s College London, Women’s Health Academic Centre<br>McManus, Richard; University of Oxford, Dept of Primary Care Health Sciences
**Primary Subject Heading**:	Obstetrics and gynaecology
Secondary Subject Heading:	Cardiovascular medicine
Keywords:	Pregnancy, Gestational Hypertension, Pre-eclampsia, Hypertension < CARDIOLOGY, Self-Monitoring, Blood Pressure

SCHOLARONE™
Manuscripts

I, the Submitting Author has the right to grant and does grant on behalf of all authors of the Work (as defined in the below author licence), an exclusive licence and/or a non-exclusive licence for contributions from authors who are: i) UK Crown employees; ii) where BMJ has agreed a CC-BY licence shall apply, and/or iii) in accordance with the terms applicable for US Federal Government officers or employees acting as part of their official duties; on a worldwide, perpetual, irrevocable, royalty-free basis to BMJ Publishing Group Ltd ("BMJ") its licensees and where the relevant Journal is co-owned by BMJ to the co-owners of the Journal, to publish the Work in this journal and any other BMJ products and to exploit all rights, as set out in our licence.

The Submitting Author accepts and understands that any supply made under these terms is made by BMJ to the Submitting Author unless you are acting as an employee on behalf of your employer or a postgraduate student of an affiliated institution which is paying any applicable article publishing charge ("APC") for Open Access articles. Where the Submitting Author wishes to make the Work available on an Open Access basis (and intends to pay the relevant APC), the terms of reuse of such Open Access shall be governed by a Creative Commons licence – details of these licences and which Creative Commons licence will apply to this Work are set out in our licence referred to above.

Other than as permitted in any relevant BMJ Author's Self Archiving Policies, I confirm this Work has not been accepted for publication elsewhere, is not being considered for publication elsewhere and does not duplicate material already published. I confirm all authors consent to publication of this Work and authorise the granting of this licence.

For peer review only

Blood pressure monitoring in high risk pregnancy to improve the detection and monitoring of hypertension (The BUMP 1&2 trials): protocol for two linked randomised controlled trials.

*Greig Dougall[1], *Marloes Franssen[1], Katherine L Tucker[1], Ly-Mee Yu,[1] Lisa Hinton, [1] Oliver Rivero-Arias [2], Lucy Abel[1], Julie Allen[1], Rebecca J Band[10], Alison Chisholm[1], Carole Crawford[1], Marcus Green[3], Sheila Greenfield[4], James Hodgkinson[4], Paul Leeson[5], Christine McCourt[6], Lucy Mackillop[7], Alecia Nickless[1], Jane Sandall[8], Mauro Santos[9], Lionel Tarassenko[9], Carmelo Velardo[9], Hannah Wilson[8], Lucy Yardley[10], +Lucy C Chappell[8], and +Richard J McManus[1]

* Joint First Authors

+ Joint Senior Authors

[1] Nuffield Department of Primary Care Health Sciences, University of Oxford, Oxford.
[2] National Perinatal Epidemiology Unit (NPEU), Nuffield Department of Population Health, University of Oxford, Oxford.
[3] Action on Pre-eclampsia, The Stables, 80 B High Street, Evesham, Worcestershire.
[4] Institute of Applied Health Research, University of Birmingham, Birmingham.
[5] Cardiovascular Clinical Research Facility, RDM Division of Cardiovascular Medicine, University of Oxford.
[6] Centre for Maternal & Child Health Research, School of Health Sciences, City, University of London, London.
[7] Nuffield Department of Women's & Reproductive Health, University of Oxford, Oxford.
[8] Department of Women and Children's Health, King's College London, St Thomas' Hospital, London.
[9] Institute of Biomedical Engineering, Department of Engineering Science, University of Oxford, Oxford.
[10] Department of Psychology, University of Southampton, Southampton, UK and School of Psychological Science, University of Bristol, Bristol.

Corresponding author: Prof Lucy Chappell

Email: lucy.chappell@kcl.ac.uk

Telephone: +44 (0) 7973 855025

Address: Department of Women and Childrens' Health. King's College London.

St Thomas' Hospital, Westminster Bridge Road, London. SE1 7EH.

Word count: 5591 Intro to Discussion (excluding title page, abstract, references, tables and figures)

Number of tables: 4 **Number of figures:** 5

For peer review only

For peer review only - http://bmjopen.bmj.com/site/about/guidelines.xhtml

ABSTRACT

Introduction:

Self-monitoring of blood pressure (BP) in pregnancy could improve the detection and management of pregnancy hypertension, whilst also empowering and engaging women in their own care. Two linked trials aim to evaluate whether BP self-monitoring in pregnancy improves the detection of raised BP during higher risk pregnancies (BUMP 1) and whether self-monitoring reduces systolic BP during hypertensive pregnancy (BUMP 2).

Methods and Analyses:

Both are multicentre, non-masked, parallel group, randomised controlled trials. Participants will be randomised to self-monitoring with telemonitoring or usual care. BUMP 1 will recruit a minimum of 2262 pregnant women at higher risk of pregnancy hypertension and BUMP 2 will recruit a minimum of 512 pregnant women with either gestational or chronic hypertension. The BUMP 1 primary outcome is the time to the first recording of raised BP by a healthcare professional. The BUMP 2 primary outcome is mean systolic BP between baseline and delivery recorded by healthcare professionals. Other outcomes will include maternal and perinatal outcomes, quality of life, and adverse events. An economic evaluation of BP self-monitoring in addition to usual care compared to usual care alone will be assessed across both study populations within trial and with modelling to estimate long-term cost-effectiveness. A linked process evaluation will combine quantitative and qualitative data to examine how BP self-monitoring in pregnancy is implemented and accepted in both daily life and routine clinical practice.

Ethics and Dissemination:

The trials have been approved by a Research Ethics Committee (17/WM/0241) and relevant research authorities. They will be published in peer-reviewed journals and presented at national and international conferences. If shown to be effective, BP self-monitoring would be applicable to a large population of pregnant women.

Trial Registration: NCT03334149 (clinicaltrials.gov)

Word Count: 277

Keywords: Pregnancy, Gestational Hypertension, Pre-eclampsia, Hypertension, Self-monitoring, Blood Pressure, Telemonitoring,

For peer review only - http://bmjopen.bmj.com/site/about/guidelines.xhtml

Strengths and limitations of these trials:

Strengths:

- Based on current literature, these will be the largest randomised controlled trials of blood pressure self-monitoring in pregnancy completed to date.
- The pragmatic trial designs, with broad inclusion criteria, will make findings of both linked studies applicable to routine antenatal care.

Weaknesses

- Data on the correct targets for self-monitored blood pressure are currently sparse.
- The trials are powered for primary and some secondary outcomes but the effect of self-monitoring on rarer secondary maternal morbidity and mortality outcomes will still be uncertain.
- Women with gestational hypertension may develop this late in pregnancy making it harder to show benefit for interventions at this time.

For peer review only

Introduction

Raised blood pressure (BP) affects approximately 10% of pregnancies worldwide, almost half of these women develop pre-eclampsia.[1] Globally, around 15% of maternal mortality is due to pre-eclampsia so early detection and prevention are paramount.[2] In the UK, inadequate management of raised BP, in particular systolic hypertension, has previously been reported as a significant contributing factor to maternal deaths and although this has improved over the past decade, pre-eclampsia remains important. [3 4]

BP self-monitoring, where an individual measures their own BP outside of the clinical setting, allows for multiple measurements providing a better estimate of the underlying BP than intermittent clinic measurements. Such self-monitoring in pregnancy could improve both the detection and subsequent management of gestational hypertensive disorders including pre-eclampsia, whilst also empowering and engaging women in their own care.[5 6]

Women who are at higher risk for raised BP in pregnancy (e.g. due to age or previous medical history) may require more frequent monitoring.[7 8] BP can rise rapidly in pregnancy and hypertension may go undetected in between antenatal visits.[2] Once raised BP is detected during pregnancy, the clinical focus is on treating the hypertension, monitoring for development of pre-eclampsia and ensuring appropriate fetal surveillance.[7] Substantial resources are currently expended in monitoring such women, from the perspective of both the woman and the National Health Service (NHS).[9] If shown to be successful, self-monitoring could provide more accurate data for clinicians to use for treatment and management strategies, safely reduce the burden of multiple clinic visits for women, free up time for midwives, and therefore be a cost-effective option. Self-monitoring is easy to accomplish and is now commonplace in adults with hypertension outside of pregnancy. [10 11]

BP self-monitoring is therefore a potentially appealing option for both the detection and management of hypertension. We have previously shown feasibility and acceptability of self-monitoring of BP in pregnancy.[5 6] This trial aims to investigate self-monitoring in two groups by undertaking two linked, parallel group, randomised trials to evaluate whether self-monitoring of BP improves the detection of raised BP during higher risk pregnancies (BUMP 1) and whether self-monitoring improves BP control in women with hypertensive pregnancy (BUMP 2).

For peer review only

Methods and Analysis

Study design and setting

The BUMP trials are two linked, multicentre, non-masked, parallel group, randomised controlled trials investigating self-monitoring of BP during pregnancy in the UK in secondary care maternity units (sites listed on https://clinicaltrials.gov/ct2/show/NCT03334149). The work is part of a larger programme of work investigating the use of self-monitoring and testing during pregnancy.

The BUMP 1 trial is a prospective non-masked randomised controlled trial of self-monitoring of BP in pregnancy for the detection of raised BP. Women at higher risk of pre-eclampsia will be recruited through antenatal clinics. The consent process for BUMP 1 will include discussion of the transition to BUMP 2 should a woman develop raised BP.

The BUMP 2 trial is a prospective non-masked randomised controlled trial of self-monitoring of BP for the management of hypertension in pregnancy in women with chronic hypertension or gestational hypertension. Women may enter this study from BUMP 1 (maintaining original randomisation) or be recruited without prior involvement. See *Figure 1* for an overview of the study design.

There will be an initial external pilot phase including up to 50 women in order to test trial procedures prior to the commencement of full recruitment.

Intervention and controls groups

Usual care

Usual care will consist of pregnant women having their BP measured by their usual antenatal care team, and initiation and/or adjustment of medication (where appropriate) based on these measurements at the discretion of the health care professional.

Self-monitoring

Women randomised to self-monitoring will be provided with a monitor validated for use in pregnancy and pre-eclampsia (Microlife WatchBP Home) and instructions for its use. Participants will be enrolled on the telemonitoring system, a text/app based system and provided with clear instructions on its use. Participants can switch between the text and app system to decrease problems with connectivity e.g. poor internet connection or phone signal. Access to the telemonitoring system was designed to have secure logins for the participants, their clinicians, and the research team. This system was developed from our pilot work with appropriate theoretical underpinnings and designed by the University of Oxford, Department of Engineering Science, and appropriate functionality testing was done by the development team to test different combinations

For peer review only - http://bmjopen.bmj.com/site/about/guidelines.xhtml

of normal and abnormal BP readings and user behaviours (e.g. poor adherence or numerous, unrequested readings) over a prolonged period followed by user testing with pregnant women.[6 12]

BUMP 1 participants will be asked to monitor their BP three times a week throughout their pregnancy. They will be instructed to sit quietly and comfortably for one minute, take two readings one minute apart and submit their second reading to the telemonitoring system. Participants will receive a guideline with colour-coded instructions (*Figure 2*). If the second reading is outside the expected range, this will automatically trigger a request for a third reading (taken after 5 minutes) and persistent high or low readings will automatically trigger a message to ask the participant to contact their local maternity unit. If women have persistently high readings, they will be asked (via the telemonitoring system) to undertake daily readings for the rest of their pregnancy.

BUMP 2 participants will be asked to monitor their BP daily throughout their pregnancy. They will be instructed to take two measures (as above) and asked to submit their second reading to the telemonitoring system. Participants will receive a guideline with colour coded instructions (*Figure 3*). High or low second readings will automatically trigger a request for a third reading (taken after 5 minutes) and persistent high or low readings will automatically trigger a message for the participant to contact their local maternity unit.

On the basis of the results of our pilot work and recent systematic review, the same thresholds will be used for self-monitoring as clinic BP to trigger action (i.e. 140/90mmHg), therefore resulting in alerts to participating women via the telemonitoring system.[6 13]

To improve adherence to the intervention participants will receive weekly motivational messages, and these messages will be sent to women in the intervention group via the telemonitoring system (via the app or text message) based on the woman's preference. These are designed to provide support and education throughout the trial and were developed during our pilot work with patient and public involvement. Messages will be selected at random from a pool of messages (approved by the ethics committee). To monitor adherence and for safety the local clinical team will also receive warnings in real time via the telemonitoring system if a participant misses submitting readings, allowing them to approach the participant to troubleshoot any potential issues.

Participants will be provided with instructions in the use of the telemonitoring system at the baseline visit. Participants will manually send their readings from the BP monitor to a centralised database, secured behind NHS firewalls, using a free SMS text message or app with web-based data entry back-up. Reminders and triggered messages will be received by participants according to a rule-based algorithm developed with the clinical team. Participants in BUMP 1 will receive a reminder after four days without submitting a reading and then one more reminder the following day if no further readings are entered. Participants in BUMP 2 will receive a reminder after two

For peer review only - http://bmjopen.bmj.com/site/about/guidelines.xhtml

missed days of readings, with a further reminder the following day if no further readings are entered.

Each participant has the right to discontinue the intervention or withdraw from the trial at any time. In addition, an investigator may withdraw a participant from the trial at any time if the investigator considers it necessary for any reason including: ineligibility (either arising during the trial or retrospectively having been overlooked at screening), an adverse event which results in inability to continue to comply with trial procedures, withdrawal of consent and loss to follow up. If a woman discontinues the intervention, withdraws or is withdrawn from the study at any point, her usual antenatal care will continue as all study procedures are additional rather than in place of usual care. Women who wish to discontinue the BP self-monitoring intervention will be asked if they are willing to participate in study follow-up. All data collected to the point of withdrawal will be retained in the study database. Unless a participant specifically withdraws consent, notes review will be conducted if possible, even where an individual has been lost to follow-up.

Study Outcomes

Outcome definitions

- The onset of hypertension/high BP will be defined as the earliest date recorded of sustained systolic BP ≥140 and/or diastolic BP ≥90 mm Hg from any community or hospital setting recorded in the notes review.
- Sustained is defined as at least two readings within 1 week (168 hours), with no minimum time between readings needing to be recorded.
- The 'earliest date recorded' will be for the second of these two readings.
- Additionally, a new prescription of antihypertensive medication for raised BP will be taken as a diagnosis of hypertension/high BP (in order to capture diagnoses where medication has been started but two raised BPs have not been recorded in the notes review for whatever reason). In this case the first date of prescription will be listed as the date of diagnosis.

Primary outcome for BUMP 1:

The primary outcome for BUMP 1 is the difference in time to a recording of raised BP by a health care professional between the usual care and self-monitoring groups.

Secondary outcomes for BUMP 1:

Secondary outcomes for BUMP 1 include analysis of the difference between usual care and self-monitoring groups as per *Table 1*:

For peer review only - http://bmjopen.bmj.com/site/about/guidelines.xhtml

For peer review only

Primary outcome for BUMP 2:

The primary outcome for BUMP 2 is the difference in mean systolic BPs, recorded by health care professionals from baseline to delivery, between usual care and self-monitoring groups.

Secondary Outcomes for BUMP 2:

Secondary outcomes for BUMP 2 include analysis of the difference between usual care and self-monitoring groups as per *Table 1*:

Participant Timeline

Members of the research team will provide a full verbal explanation and written description of the trial to women who meet the inclusion criteria (*Table 2 & 3*). The woman will be given sufficient time to consider the information, and to decide whether she will participate in the trial. Written informed consent will be sought from the woman and taken by an appropriately trained healthcare professional. Eligibility will be assessed and baseline data, including all demographics, maternal demographics and patient questionnaires (completed by the woman), will be entered on a web-based database by members of the research team. Participants will be randomised and instructed in the use of self-monitoring and provided with a BP monitor or instructed to follow usual care. Women randomised to usual care will not be prevented from self-monitoring although we will ask all women at final follow-up if they have self-monitored during the trial. Participants on both arms will follow their routine care clinic visits until delivery. Participants will be contacted at around 30 weeks' gestation (2 weeks after baseline if participants were recruited after 30 weeks) and asked either in person/over the phone/by post/ or via email to complete patient questionnaires. The remainder of antenatal care, in particular the timing and mode of delivery, will be left to the discretion of the responsible clinician. A medical notes review will be undertaken following the primary discharge for mother and baby. If a woman and/or her baby is still in hospital at 2 months from delivery or 2 months from her estimated date of delivery (whichever is longer), the admission time will be censored and data collected up to that point. Self-monitoring of BP will finish at admission in established labour or the end of pregnancy, whichever is sooner and participants will continue with standard postnatal care. Participants will be contacted at around 8 weeks post-delivery and asked either in person/over the phone/by post/ or via email to complete patient questionnaires and arrange for collection of the loaned BP monitor if not returned at delivery. A schedule of participant enrolment, interventions and assessments in the trial is shown in *Table 4* and a flowchart of study visits is shown in *Figures 4 & 5.*

For peer review only - http://bmjopen.bmj.com/site/about/guidelines.xhtml

Sample size considerations

BUMP 1:

The sample size was determined using a two stage simulation process which modelled how many women would be expected to develop hypertension and how long time to detection would take in these women, using data from our pilot work in the BUMP study. Assuming 16% of women develop hypertension, and a standard deviation (SD) of 40 days to detection of raised BP in both groups, a sample size of 2262 (1131 per group) will allow detection of an effect size of 12 day's difference in time to detection of raised BP in pregnancy between self-monitoring and control groups (the primary outcome of BUMP 1), with 90% power and 5% level of significance (2-sided) and assuming a 15% attrition rate. If the SD is 45 days, then this sample size will allow detection of a difference of 14 days with more than 90% power and if the SD is 50 days then it will be sufficient to detect a difference of 16 days in time to detection of raised BP in pregnancy also with 90% power. Of the 2262 women recruited to BUMP 1, around 362 women are expected to develop hypertension. We will recruit a minimum of 2262 women to ensure adequate power. The simulation was carried out using R 3.1.2. (https://www.r-project.org/).

BUMP 2:

A sample size of 256 per group will be sufficient to detect a 5mmHg difference in systolic BP between groups, accounting for 15% attrition and an SD of 16mmHg, based on data from the BUMP pilot and PELICAN studies, inflated from 14mmHg because BUMP 2 will include hypertensive women from BUMP 1 and those with chronic or gestational hypertension not previously randomised in BUMP 1.[6 14] The sample size was calculated using NCSS PASS v12.0.

Women randomised in BUMP 1 who develop hypertension will remain in their randomisation groups and move seamlessly into the BUMP 2 trial. This will occur automatically via the telemonitoring system and their inclusion in BUMP 2 assessed retrospectively. The precise numbers and proportion of women migrating from BUMP 1 to BUMP 2 will not be fixed. We will aim to recruit a minimum of 512 women directly into the BUMP 2 trial in addition to those that migrate in order to ensure adequate power: women who have chronic hypertension or have developed gestational hypertension and are not taking part in the BUMP 1 trial will be randomised to either usual care using clinic BP to guide treatment or BP self-monitoring with telemonitoring and automated feedback.

Both trials will be undertaken in approximately 15 maternity units in England to achieve the sample size in the anticipated time-frame. Our previous pilot trial has confirmed that women and clinicians are willing to participate in this type of randomised controlled trial and we have used recruitment

For peer review only - http://bmjopen.bmj.com/site/about/guidelines.xhtml

estimates from this trial, together with trial management experience and expertise of the Co-Investigator Group to inform strategies to meet the required target size.[6]

Data management

An online clinical data management system will be used, with all clinical data entered directly onto the electronic CRFs and stored on a study specific database. Paper back-up forms will be available if the online database cannot be accessed. Data collected on paper forms will be entered on to the database at the earliest opportunity, by single data entry. Patient-completed questionnaires will be completed directly on to the system by the participant wherever possible, with manual single data entry of paper copies if required. Telemonitored mHealth data is stored on a centralised database, secured behind NHS firewalls, designed and maintained by the University of Oxford, Department of Engineering Science.

Randomisation

Women who agree to participate in the trial (BUMP 1 or BUMP 2) will be randomised on a 1:1 ratio either to BP self-monitoring or usual care by the recruiting researcher. An independent statistician will generate a randomisation sequence list for each trial, using permutated varying blocks and stratified by recruitment site and parity. The generated schedules were then imported to the randomisation module within the online data management system for site to carry out the randomisation. Women will be randomised by the recruiting researcher using the online data management system. Women who develop hypertension during BUMP 1 and migrate to BUMP 2 will stay in their original randomisation group. A manual telephone based back-up randomisation system will be used in the event the online system is not available.

Statistical analysis

General consideration for both trials:

The primary statistical analysis will be carried out on the basis of intention-to-treat (ITT). Thus, after randomisation participants will be analysed according to their allocated treatment group irrespective of what treatment they actually receive. We will endeavour to obtain full follow-up data on every participant to allow full ITT analysis, but we will inevitably experience the problem of missing data due to withdrawal, loss to follow up, or nonresponse questionnaire items. The results from the trial will be prepared as comparative summary statistics (e.g. difference in means) with 95% confidence intervals. The study results will be reported in accordance with the CONSORT 2010

For peer review only - http://bmjopen.bmj.com/site/about/guidelines.xhtml

statements and full detailed statistical analysis plans will be prepared before the final analysis by an independent statistician.

BUMP 1:

The primary analysis will determine whether there is a difference in the time to diagnosis between the randomised groups (usual care vs self-monitoring) and will be performed using a two-part model, such as the hurdle model.[15] The model takes into account participants who developed a raised BP or not and the time to raise BP, adjusting for stratification factors. This method has the advantage that all women who are recruited to BUMP 1 will contribute to the primary analysis. Continuous secondary outcomes, such as birth weight and length of stay, will be analysed by means of regression method adjusting for stratification factors. Binary secondary outcomes, such as the development of severe hypertension, development of any complications, and stillbirth rate, will be analysed by means of a log binomial model.

BUMP 2:

An ITT analysis will include women recruited to BUMP 1 who become hypertensive and women recruited de novo to BUMP 2 with GH and CH analysed separately. The primary analysis for BUMP 2 will in each case compare BPs between the intervention and control groups. Analysis of the BP outcome will be by means of a linear mixed effects model which can accommodate data where participants have repeated measurements, and also accounts for missing data (assuming data are missing at random (MAR)). Each of a participant's BP measures will be modelled by means of a linear mixed effects model, including random effects for participants and assuming an unstructured variance covariance matrix between measurements from the same participant. Time of BP measurement and randomised group, as well as their interaction term, will be included as fixed effects in the model. A fixed effect will also be included for whether the participant was from BUMP 1 or not in the GH analysis. As a sensitivity analysis inclusion from BUMP 1 or not will be tested as a moderator of the treatment effect. By means of appropriate contrasts, the difference in mean BP of participants in the randomised groups from both BUMP 1 and de novo recruitment will be compared.

BP load and area under the curve will be analysed as continuous outcomes by means of a linear regression model, adjusting for whether the participant originated from BUMP 1. Continuous secondary outcomes, such as birth weight and length of stay, will be analysed by means of regression model. Binary secondary outcomes, such as the development of severe hypertension, incidence of complications and incidence of stillbirth, will be analysed by means of a log binominal model.

For peer review only - http://bmjopen.bmj.com/site/about/guidelines.xhtml

Sub-Group Analyses:

For both BUMP 1 and BUMP 2 pre-specified subgroups include eligibility for aspirin prescription to prevent pre-eclampsia (As per NICE); gestational age at baseline (≤20 weeks' vs >20 weeks), parity (0 vs ≥1). In addition, a sensitivity analysis will compare those women with GH that are recruited de novo into BUMP 2 with those that migrate from BUMP 1.

Economic evaluation

The economic evaluation component aims to determine whether BP self-monitoring in addition to usual care represents value for money compared to usual care alone in a population of higher risk pregnant women (BUMP 1) and women with a diagnosis of hypertension during pregnancy (BUMP 2). Two separate within-trial cost-effectiveness analyses will be carried out alongside BUMP 1 and BUMP 2. In addition, a decision analytic model will be built to determine the long-term cost-effectiveness of self-monitoring in pregnancy. A UK National Health Service (NHS) perspective will be adopted in all analyses.

The within-trial cost-utility analyses will use individual patient-level data with a time horizon up to two months following delivery. The main health outcome measure in the economic evaluation will be maternal quality-adjusted life years (QALYs) over the trial period. The calculation of a QALY profile for each woman will be informed using data from the health-related quality of life instrument EQ-5D-5L that will be collected at baseline, 30 weeks' gestation and eight weeks after birth.[16] A linear change between utility measures at each time period will be assumed when deriving an individual's QALY profile. Relevant healthcare resource use during pregnancy for mothers and their fetuses will include antenatal, intrapartum and postnatal care. Antenatal care utilisation will include primary and secondary care visits including hospital admissions. Intrapartum and related postnatal care before primary hospital discharge will include major procedures undertaken, length of stay and transfers for mothers and their babies. Primary and secondary care resource utilisation during the trial will be collected using case note review including routine and additional clinic visits to midwives and GPs. Unit costs will be extracted from primarily national sources.[17-19] The cost of using an automated monitor along with the telemonitoring system will be estimated and included in the cost analysis of the self-monitoring group in each trial. Mean maternal QALYs and costs (and associated uncertainty) will be estimated parametrically in the intervention and usual care group in each of the trials. If necessary, we will use multiple imputation methods to deal with missing EQ-5D-5L and resource use data over the trial period and will follow current guidance to conduct and report such analysis.[20]

The long-term decision analytical model will include a decision tree to represent women's pathways up to the first eight weeks post-delivery and a Markov model representing the history of disease of

For peer review only - http://bmjopen.bmj.com/site/about/guidelines.xhtml

women with raised BP after that point, using annual cycles. The structure of the model will be based on models identified via a systematic review of previous economic evaluations of hypertensive patients in pregnancy (currently in progress with PROSPERO number: 123881). Observed risk factors, quality of life and health care resource utilisation for randomised participants in BUMP 1 and BUMP 2 will be used to inform the characteristics of a hypothetical cohort entering the model for each treatment pathway. Transition probabilities indicating movement across health states will be informed by cardiovascular risk predictions modified using the literature on risk following hypertensive pregnancy. Costs incurred annually in each health state will be obtained from the literature differentiating between acute and chronic disease health states. Health-related quality of life associated with each health state in the model will also be obtained from the literature.

Both the within-trial cost-effectiveness analysis and long-term model will be reported using current reporting standards for economic evaluations.[21] Mean differences in costs and QALYs between intervention and usual care arms in each trial and the model will be combined to generate an incremental cost-effectiveness ratio (ICER), presented as cost per QALY gained. Uncertainty around ICERs will be handled using confidence intervals (if appropriate) and cost-effectiveness acceptability curves. Uncertainty in the long-term model will be assessed using probabilistic sensitivity analysis. Recent guidance will also be used to report and interpret the results from the decision analytic model.[22]

Process Evaluation (qualitative and quantitative)

Alongside the trial we will undertake a process evaluation to assess both quantitative (including the outcomes listed in *Table 1* above) and qualitative (see below) aspects. Intervention fidelity will be assessed for women and participating professionals. This will include for the women: examining persistence with self-monitoring and comparing submitted readings and monitor downloads for a sub-set of women. For the professionals, a staff survey will assess practice in terms of utilisation of self-monitored BP readings during the trial.

In addition to the quantitative process evaluation, a qualitative study will be conducted within the trial to understand how BP self-monitoring in pregnancy is implemented and its acceptability in daily life and routine clinical practice. In-depth interviews will be completed with a sample of participating women in both BUMP 1 and BUMP 2 and healthcare professionals.[23]

Participating women: The study aims to include approximately 40 pregnant women across five sites, including a sample of up to eight women who decide not to take part or discontinue from the intervention and are willing to be interviewed.[24] The interviews with women will look to understand

For peer review only - http://bmjopen.bmj.com/site/about/guidelines.xhtml

real-life implementation issues with BP self-monitoring. Women who have consented to contact at main trial enrolment will be contacted by phone/email/in person regarding the interviews and provided with a patient information sheet (PIS); they will be consented separately prior to the start of the interview. Additionally, an ethnographic study will follow a sample of 10-15 women who monitor their BP.[25] This ethnographic study will include longitudinal interviewing and non-participatory observations to understand in detail how women engage with home monitoring over time and whether and how home BP readings are incorporated into clinical encounters.[26 27]

Healthcare professionals: Interviews will be conducted with 30-40 midwives and obstetricians who are involved in BUMP 1 and BUMP 2.[24] The aim is to understand how self-monitoring is operationalised in practice and provide key information for the process evaluation.

Detailed field notes will be taken of observations. Interviews will be audio recorded so that content can be transcribed. Data collection and analysis will be guided by theory, including social cognitive theory, a "technology in practice" perspective and normalisation process theory as interpretative frameworks to understand how the trial intervention 'fits' within existing practices and systems, and how it becomes embedded (or not) in women's lives and healthcare professionals' routine work.[28 29 30 31] Inductive and deductive approaches to categorising and coding the data will be combined, drawing on *a priori* theoretical concepts (including, for example, self-efficacy, unexpected use of technology, and cognitive participation) while remaining sensitive to themes that emerge from the data themselves. An iterative analytic process will be employed to map the range of phenomena and identify associations between themes with the aim of illuminating the mechanisms that underpin the outcomes of the intervention.[32] QSR NVivo will be used to support the organisation and retrieval of the qualitative data.[33]

Data Monitoring

Regular monitoring will be performed by the trial team according to Good Clinical Practice and Clinical Trial Unit standard operating procedures. Data will be evaluated for compliance with the protocol and accuracy in relation to source documents. Following written standard operating procedures, the monitors will verify that the clinical trial is conducted and data are generated, documented and reported in compliance with the protocol, GCP and the applicable regulatory requirements.

A Trial Steering Committee (TSC) and Data Monitoring Committee (DMC) will be convened and review trial progress every 6 months. The TSC will provide overall supervision of the trial and ensure its conduct is in accordance with the principles of GCP and the relevant regulations. The TSC will agree the trial protocol and provide advice to the investigators on all aspects of the trial. The TSC will

For peer review only - http://bmjopen.bmj.com/site/about/guidelines.xhtml

include members who are independent of the investigators, in particular an independent chairperson. The independent DMC will inform the TSC regarding the accruing trial and safety data, to ensure trial site staff and participants are aware of any relevant safety information, and to advise the TSC regarding the appropriateness of continuation of the trial. Any consideration of the need for termination of the trial will be on the advice of the DMC and TSC who will consider the need for prospective criteria for termination.

Harms/ Potential Risks

During these randomised controlled trials participants will continue to receive "usual care" regardless of randomisation group, and thus we anticipate that the potential risks are low. Particular issues include the possibility of increased anxiety due to the study. Training of participants will cover repeated measurements in the case of unusually high or low readings. The participant guideline/booklet will give clear advice to women to contact the antenatal care team or other healthcare professional (e.g. General Practitioner (GP)) in the case of persistent high or low readings. The telemonitoring system will automatically provide this advice when high or low readings are sent in. Women will continue to be seen by their clinical teams (midwives/ GPs/ obstetricians) at frequencies chosen by their clinicians throughout, regardless of randomisation group.
Participants will be advised to seek immediate medical help if they experience any symptoms of pre-eclampsia, regardless of their BP readings.

It is not anticipated that the study intervention (self-monitoring of BP) should result in any adverse events (AEs) but collection of such events will be included in case such events occur so that they can be considered for causal links to the study. Only AEs that are clinically judged (by the supervising site Principal Investigator) as being caused by the trial intervention will be reported to the Clinical Trials Unit to raise appropriately to the sponsor/Research Ethics Committee that gave favourable opinion. In addition, any maternal death or stroke, fetal loss or neonatal death will be reported to the data monitoring committee regardless as to whether they are judged related. Side effects as stated in the British National Formulary will not be reported as AEs.

For peer review only - http://bmjopen.bmj.com/site/about/guidelines.xhtml

Ethics and Dissemination

Ethics approval and consent to participate

The protocol, informed consent form, participant information sheet and all other participant-facing material have been approved by the Research Ethics Committee (West Midlands - South Birmingham: ref 17/WM/0241), host institution(s) and Health Research Authority. Any subsequent protocol amendments will be agreed with both sponsor and ethics committee prior to implementation. The study sponsor reviewed and ensured all indemnity and insurance requirements for the trial were in place prior to the start of recruitment which would operate in the event of any participant suffering harm as a result of their involvement in the research. NHS indemnity operates in respect of the clinical treatment which is provided. Participants will provide written informed consent prior to enrolment. An independent trial steering group will monitor study progress assisted by an independent data monitoring committee and will periodically review the study.

Confidentiality

The trial staff will ensure that the participants' anonymity is maintained in the trial database. The participants will be identified only by a participant identification number on all trial documents and any electronic database. Women's identifiers (names, address, phone number) will be held securely and separately from the Case Report Forms where they are needed to contact participants on an ongoing basis, e.g. for follow-up and return of BP monitor. Access to these data will be strictly on a need to know basis. All documents will be stored securely and only accessible by trial staff and authorised personnel. The study will comply with the Data Protection Act and General Data Protection Regulation (GDPR) 2018, which requires data to be anonymised as soon as it is practical to do so.

Patient and Public Involvement

Methods of patient approach, patient information sheet and consent form were all reviewed by patient representatives prior to formal approval. The relevant group for women with pre-eclampsia, "Action on Pre-Eclampsia" are formally represented through their Chief Executive Officer (MG) who is a co-investigator. The Trial Steering Committee includes an independent patient representative who is jointly responsible for overseeing the conduct of the trial with the rest of the committee.

Dissemination

All research outputs from this work will be published in peer-reviewed journals, presented at scientific conferences and lay and social media (e.g. Twitter, blogs). All trial data is the property of the Chief Investigator and will be stored until the end of the study, database lock and final analysis.

For peer review only - http://bmjopen.bmj.com/site/about/guidelines.xhtml

All research data and documentation will be appropriately archived for at least five years. 'Patient friendly' study summary documents and infographics will be made available to all participants at the end of the trial via the study website.

Discussion

This article describes the protocol for the BUMP trials. These randomised controlled trials will assess whether self-monitoring of BP during pregnancy, over and above usual care, can improve the detection of raised BP during pregnancy and whether self-monitoring can improve the control of BP in pregnancy hypertension.

Additional work will show whether self-monitoring BP is acceptable to pregnant women and their health care professionals and if it is cost-effective compared to usual care.

The results of the trial will provide data on the effects of self-monitoring and telemonitoring, over and above usual care, in pregnant women at risk for pre-eclampsia and women with hypertension. If self-monitoring of BP is found to be successful in the detection of hypertension in pregnancy and control of BP in pregnancy hypertension, then it would be applicable to a large group of pregnant women in the UK but also worldwide.

Current trial status

The trials commenced recruitment on 11th September 2017 (external pilot) with main recruitment starting on (22nd November 2017) and recruitment is planned to continue until end of September 2019. Data collection will continue to mid-2020.

ABBREVIATIONS

BP: Blood Pressure; GH: Gestational Hypertension; CH: Chronic Hypertension; EQ-5D-5L: European Questionnaire 5 Dimensions; GP: General Practitioner; mmHg: Millimetre of mercury; NHS: National Health Service; NICE: National Institute for Health and Care Excellence; PC-CTU: Primary Care – Clinical Trials Unit

For peer review only - http://bmjopen.bmj.com/site/about/guidelines.xhtml

DECLARATIONS

Competing interests

RJM has previously received BP monitors from Omron for research purposes. The BP monitors for the current trials were purchased from the manufacturer (Microlife) at commercial prices. The authors declare no conflicts of interest.

Funding

This work is funded from a National Institute for Health Research (NIHR) Programme grant for applied research (RP-PG-1209-10051) and an NIHR Professorship awarded to RJM (NIHR-RP-R2-12-015). RJM and KT receives funding from the National Institute for Health Research (NIHR) Collaboration for Leadership in Applied Health Research and Care Oxford at Oxford Health NHS Foundation Trust. JS is a National Institute for Health Research (NIHR) Senior Investigator and supported by the National Institute for Health Research (NIHR) Collaboration for Leadership in Applied Health Research and Care South London (NIHR CLAHRC South London) at King's College Hospital NHS Foundation Trust. Service support costs will be administered through the NIHR Clinical Research Network. The views expressed in this publication are those of the authors and not necessarily those of the NHS, the NIHR or the Department of Health and social care.

Authors' contributions

RJM had the original idea and applied for funding with KT, LC, LMY, LH, ORA, CC, MG, SG, JH, PL, CM, LM, JS, LT and LY. MF, GD, RJM, LC and KT wrote the first draft. AN and LMY provided the sample size calculations and statistical analysis section. RJM, KT, LH, RB, LY, MS and CV worked on developing the intervention. CC and HW are coordinating research midwives. LH and AC provided the qualitative section. ORA and LA provided the economic evaluation section. GD is the trial manager (previously MF) and JA is the senior trial manager. All authors subsequently critically edited the manuscript. RJM will be guarantor for the manuscript. All authors have read and approved the final manuscript.

Acknowledgements

The authors acknowledge the support of the Primary Care Clinical Trials Unit; Margaret Glogowska, Jacqui Williams and Tricia Carver have served as patient representatives for the trial management and steering groups. Additional members of the trial steering group are Laura Magee (chair), Jim Thornton, John Norrie and Tim Coleman. Members of the data monitoring committee are Nigel Simpson (chair), Julia Sanders and Miliça Bucknall. The sponsor and funder had no role in the study design, writing of

For peer review only - http://bmjopen.bmj.com/site/about/guidelines.xhtml

the paper; or the decision to submit this protocol for publication, which was made jointly by the authors who have all approved the final manuscript.

Protocol Version: **V**2.0 26.11.2018

Trial Sponsor: University of Oxford

Contact name: Ms Heather House

Address: Clinical Trials and Research Governance, Joint Research Office, Block 60, Churchill Hospital, University of Oxford, Oxford, OX3 7LE

Email: ctrg@admin.ox.ac.uk

For peer review only

For peer review only - http://bmjopen.bmj.com/site/about/guidelines.xhtml

FIGURES AND TABLES

Table 1: Secondary Outcomes for BUMP 1 and BUMP 2

**Maternal Outcomes**
• Severe hypertension (systolic BP ≥160mmHg and/or diastolic BP ≥110mmHg)
• Pre-eclampsia or Gestational Hypertension
• Serious maternal complications including eclampsia, transient ischemic attack or stroke, pulmonary oedema, renal failure, blood transfusion, HELLP syndrome, Liver Involvement, Haematological Involvement and death
• Onset of labour
• Change in maternal quality of life from randomisation (measured using the EQ-5D-5L questionnaire)[16]
• Diastolic blood pressure*
• Area under the blood pressure curve*
• Proportion of systolic readings above 140mmHg*
**Perinatal Outcomes**
• Stillbirths
• Early neonatal deaths
• Gestation at delivery
• Mode of delivery
• Birth weight of the baby, including centile
• Small for gestational age infants (<10th and <3rd centile)
• Neonatal unit admissions, including length of stay
**Process Outcomes**
• Change in health behaviours (questionnaire)
• Fidelity to monitoring schedule
• Change in State Trait Anxiety Inventory short form (STAI-6) anxiety questionnaire[34]
• Health service costs
• Cost per maternal quality-adjusted life year gained over trial period
• Change in adherence to medication (MARS questionnaire)*[35]
• Qualitative data gathered from participating women and healthcare professionals

Outcomes common to both trials apart from *only Assessed on BUMP 2 Participants

For peer review only

For peer review only - http://bmjopen.bmj.com/site/about/guidelines.xhtml

Table 2: BUMP 1 Inclusion and Exclusion Criteria

**Inclusion Criteria:**
• Participant is willing and able to give informed consent for participation in the trial
• Pregnant woman, aged 18 years or above between 16^{+0} to 24^{+0} weeks
• Able and willing to comply with trial requirements
• Willing to allow her GP and consultant, if appropriate, to be notified of participation in the trial
• At higher risk for hypertension in pregnancy / pre-eclampsia defined as one or more of the following risk factors:<br>○ Age 40 years or older<br>○ Nulliparity<br>○ Pregnancy interval of more than 10 years<br>○ Family history of pre-eclampsia<br>○ Previous history of pre-eclampsia or gestational hypertension<br>○ Body mass index 30 kg/m2 or above at booking<br>○ Chronic kidney disease<br>○ Twin pregnancy<br>○ Pre-pregnancy diabetes (Type 1 or 2)<br>○ Autoimmune Disease (e.g. systemic lupus erythematosus or antiphospholipid disease)
**Exclusion Criteria:**
• Chronic Hypertension

For peer review only

Table 3: BUMP 2 Inclusion and Exclusion Criteria

Inclusion Criteria:
• Women with chronic hypertension (defined as sustained systolic BP≥140 mmHg and/or diastolic BP≥90 mmHg, present at booking or before 20 weeks gestation, or receiving antihypertensive treatment outside pregnancy and/or at time of referral), recruited up to 37^{+0} weeks' gestation. *OR* • Women with gestational hypertension after 20 weeks gestation (defined as sustained systolic BP≥140 mmHg and/or diastolic BP≥90 mmHg), recruited at 20^{+0} to 37^{+0} weeks' gestation. *AND*
• Participant is willing and able to give informed consent for participation in the trial.
• Woman aged 18 years or above
• Willing to allow her GP and consultant, if appropriate, to be notified of participation
**Exclusion Criteria:**
• Anticipated inpatient admission considered likely to lead to imminent delivery (within the next 48 hours)

For peer review only

For peer review only - http://bmjopen.bmj.com/site/about/guidelines.xhtml

Table 1: Schedule of interventions and assessments.

	STUDY PERIOD (16 weeks to 8 weeks post-delivery)			
**Study Visit**	**Trial Entry/Baseline**	**Antenatal Follow Up**	**Notes Review**	**Postnatal Follow Up**
**TIMEPOINT**	***20±4 Weeks' Gestation***	**30±3 Weeks' *Gestation***	***40+4 Weeks' (Post Delivery)***	***40+8 Weeks' (Post Delivery)***
**Informed Consent**	X			
**Eligibility Assessment**	X			
**Demographics; Including age, race, education**	X			
**Maternal Demographics; including gestation, EDD and Parity**	X			
**Patient Questionnaires; Including EQ-5D-5L and STAI**	X	X		X
**Randomisation**	X			
**Recording Home Blood Pressure**	**Self-Monitoring group will record and submit reading between baseline and delivery**			
**Recording Clinic Blood Pressures**			X	
**Maternal and Perinatal Outcomes**			X	
**Secondary Outcome Data**			X	
**Return of BP Monitor**			**BP Monitors will be returned at/or post delivery**	

For peer review only

For peer review only - http://bmjopen.bmj.com/site/about/guidelines.xhtml

Figure Legend:

Figure 1: Study Design

Abbreviations: HT – Hypertension, NT – Normotension, CH - Chronic Hypertension, GH – Gestational Hypertension

Figure 1: Blood Pressure Interpretation Chart for BUMP 1

Figure 2: Blood Pressure Interpretation Chart for BUMP 2

Figure 3: BUMP 1 Study Visit Chart

Figure 4: BUMP 2 Study Visit Chart

For peer review only

For peer review only - http://bmjopen.bmj.com/site/about/guidelines.xhtml

References

1. Khan KS, Wojdyla D, Say L, et al. WHO analysis of causes of maternal death: a systematic review. *Lancet* 2006;367(9516):1066-74. doi: 10.1016/S0140-6736(06)68397-9 [published Online First: 2006/04/04]
2. Douglas KA, Redman CW. Eclampsia in the United Kingdom. *BMJ* 1994;309(6966):1395-400. [published Online First: 1994/11/26]
3. Cantwell R, Clutton-Brock T, Cooper G, et al. Saving Mothers' Lives: Reviewing maternal deaths to make motherhood safer: 2006-2008. The Eighth Report of the Confidential Enquiries into Maternal Deaths in the United Kingdom. *BJOG* 2011;118 Suppl 1:1-203. doi: 10.1111/j.1471-0528.2010.02847.x [published Online First: 2011/03/05]
4. Knight M, Tuffnell, D., Kenyon, S., Shakespeare, J., Gray, R., & Kurinczuk, J. J. Saving lives, improving mothers' care: Surveillance of maternal deaths in the UK 2011-13 and lessons learned to inform maternity care from the UK and Ireland. Confidential Enquiries into Maternal Deaths and Morbidity 2009-13. *National Perinatal Epidemiology Unit, University of Oxford* 2015
5. Hinton L, Tucker KL, Greenfield SM, et al. Blood pressure self-monitoring in pregnancy (BuMP) feasibility study; a qualitative analysis of women's experiences of self-monitoring. *BMC Pregnancy Childbirth* 2017;17(1):427. doi: 10.1186/s12884-017-1592-1 [published Online First: 2017/12/21]
6. Tucker KL, Taylor KS, Crawford C, et al. Blood pressure self-monitoring in pregnancy: examining feasibility in a prospective cohort study. *BMC Pregnancy Childbirth* 2017;17(1):442. doi: 10.1186/s12884-017-1605-0 [published Online First: 2017/12/30]
7. Hypertension in pregnancy: diagnosis and management (NICE Guideline 107): National Institute for Health and Care Excellence; 2010 [Available from: https://www.nice.org.uk/guidance/cg107 accessed 20 November 2018.
8. Antenatal care for uncomplicated pregnancies (NICE Guideline 62): National Institute for Health and Care Excellence; 2008 [Available from: https://www.nice.org.uk/guidance/cg62 accessed 20 November 2018.
9. Maternity Services in England: The National Audit Office. The Stationery Office, Department of Health. London., 2013.
10. Baral-Grant S, Haque MS, Nouwen A, et al. Self-Monitoring of Blood Pressure in Hypertension: A UK Primary Care Survey. *Int J Hypertens* 2012;2012:582068. doi: 10.1155/2012/582068 [published Online First: 2011/10/21]
11. Bray EP, Holder R, Mant J, et al. Does self-monitoring reduce blood pressure? Meta-analysis with meta-regression of randomized controlled trials. *Ann Med* 2010;42(5):371-86. doi: 10.3109/07853890.2010.489567 [published Online First: 2010/05/28]
12. Band Rea. Intervention planning and modification of the BUMP intervention: a digital intervention for the early detection of raised blood pressure in pregnancy. *Pilot Feasibility Stud* In Press
13. Tucker KL, Bankhead C, Hodgkinson J, et al. How Do Home and Clinic Blood Pressure Readings Compare in Pregnancy? *Hypertension* 2018;72(3):686-94. doi: 10.1161/HYPERTENSIONAHA.118.10917 [published Online First: 2018/10/26]
14. Chappell LC, Duckworth S, Seed PT, et al. Diagnostic Accuracy of Placental Growth Factor in Women With Suspected Preeclampsia A Prospective Multicenter Study. *Circulation* 2013;128(19):2121-31. doi: 10.1161/Circulationaha.113.003215
15. Cragg JG. Some Statistical Models for Limited Dependent Variables with Application to the Demand for Durable Goods. *Econometrica* 1971;39(5):829-44.
16. Herdman M, Gudex C, Lloyd A, et al. Development and preliminary testing of the new five-level version of EQ-5D (EQ-5D-5L). *Qual Life Res* 2011;20(10):1727-36. doi: 10.1007/s11136-011-9903-x [published Online First: 2011/04/12]

For peer review only - http://bmjopen.bmj.com/site/about/guidelines.xhtml

17. British National Formulary 2017 [Available from: Available at https://bnf.nice.org.uk/ accessed (Last accessed: 22 January 2019.
18. Curtis LaB, A. Unit Costs of Health and Social Care. University of Kent, Personal Social Services Research Unit. 2017 [Available from: Available at https://www.pssru.ac.uk/project-pages/unit-costs/unit-costs-2017/ accessed (Last accessed: 17 January 2019).
19. NHS reference cost 2017-18 [Available from: Available at https://improvement.nhs.uk/resources/reference-costs/#rc1718 accessed (Last accessed: 17 January 2019).
20. Faria R, Gomes M, Epstein D, et al. A guide to handling missing data in cost-effectiveness analysis conducted within randomised controlled trials. *Pharmacoeconomics* 2014;32(12):1157-70. doi: 10.1007/s40273-014-0193-3 [published Online First: 2014/07/30]
21. Husereau D, Drummond M, Petrou S, et al. Consolidated Health Economic Evaluation Reporting Standards (CHEERS)--explanation and elaboration: a report of the ISPOR Health Economic Evaluation Publication Guidelines Good Reporting Practices Task Force. *Value Health* 2013;16(2):231-50. doi: 10.1016/j.jval.2013.02.002 [published Online First: 2013/03/30]
22. Caro JJ, Briggs AH, Siebert U, et al. Modeling good research practices--overview: a report of the ISPOR-SMDM Modeling Good Research Practices Task Force--1. *Value Health* 2012;15(6):796-803. doi: 10.1016/j.jval.2012.06.012 [published Online First: 2012/09/25]
23. Jamshed S. Qualitative research method-interviewing and observation. *J Basic Clin Pharm* 2014;5(4):87-8. doi: 10.4103/0976-0105.141942 [published Online First: 2014/10/16]
24. Sandelowski M. Sample size in qualitative research. *Res Nurs Health* 1995;18(2):179-83. [published Online First: 1995/04/01]
25. Reeves S, Kuper A, Hodges BD. Qualitative research methodologies: ethnography. *BMJ* 2008;337:a1020. doi: 10.1136/bmj.a1020 [published Online First: 2008/08/09]
26. Hermanowicz JC. The Longitudinal Qualitative Interview. *Qual Sociol* 2013;36(2):189-208. doi: 10.1007/s11133-013-9247-7
27. Williams J. Nonparticipant observation2008:561-62.
28. Timmermans S, Berg M. The practice of medical technology. *Sociol Health Illn* 2003;25:97-114. [published Online First: 2003/09/23]
29. Yardley L, Morrison L, Bradbury K, et al. The person-based approach to intervention development: application to digital health-related behavior change interventions. *J Med Internet Res* 2015;17(1):e30. doi: 10.2196/jmir.4055 [published Online First: 2015/02/03]
30. May CR, Mair FS, Dowrick CF, et al. Process evaluation for complex interventions in primary care: understanding trials using the normalization process model. *BMC Fam Pract* 2007;8:42. doi: 10.1186/1471-2296-8-42 [published Online First: 2007/07/26]
31. F. S. Understanding and Using Health Experiences: improving patient care: Observing interactions as an approach to understanding patients' experiences: Oxford Oxford University Press 2013.
32. Pope C, Ziebland S, Mays N. Qualitative research in health care. Analysing qualitative data. *BMJ* 2000;320(7227):114-6. doi: 10.1136/bmj.320.7227.114 [published Online First: 2000/01/22]
33. Ltd QIP. NVivo qualitative data analysis software2018.
34. Marteau TM, Bekker H. The development of a six-item short-form of the state scale of the Spielberger State—Trait Anxiety Inventory (STAI). 1992;31(3):301-06. doi: 10.1111/j.2044-8260.1992.tb00997.x
35. Horne R, Weinman J. Patients' beliefs about prescribed medicines and their role in adherence to treatment in chronic physical illness. *J Psychosom Res* 1999;47(6):555-67. doi: Doi 10.1016/S0022-3999(99)00057-4

For peer review only - http://bmjopen.bmj.com/site/about/guidelines.xhtml

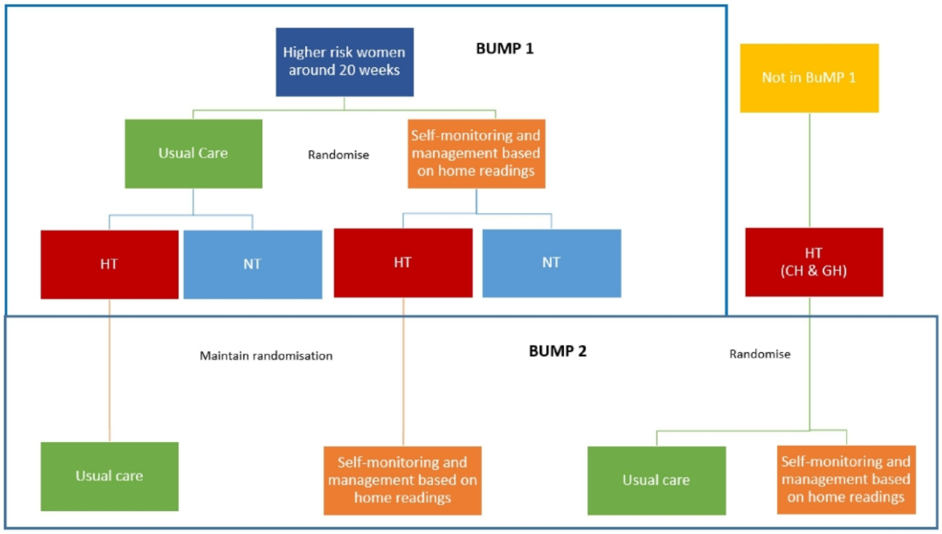

Figure 1: Study Design
HT – Hypertension, NT – Normotension, CH - Chronic Hypertension, GH – Gestational Hypertension

274x162mm (300 x 300 DPI)

LEVEL	BLOOD PRESSURE /mmHg	ACTION
**HIGH**	SYS 150 or more<br><br>OR<br><br>DIA 100 or more	Your blood pressure is high<br><br>Sit quietly for 5 minutes then measure it again and send in the reading.<br><br>Contact your maternity unit for urgent assessment today (within 4 hours) and continue to monitor your BP daily.
**RAISED**	SYS 140-149<br><br>OR<br><br>DIA 90-99	Your blood pressure is raised<br><br>Sit quietly for 5 minutes then measure it again and send in the reading.<br><br>If your repeated reading is raised please contact your maternity unit within 24 hours and continue to monitor your BP daily.
**HIGH NORMAL**	SYS 135-139<br><br>OR<br><br>DIA 85-89	Your blood pressure is normal but moving towards the raised threshold<br><br>Sit quietly for 5 minutes then measure it again and send in the reading.<br><br>If your repeat reading is still high-normal, please monitor your blood pressure daily.
**NORMAL**	SYS 110-134<br><br>OR<br><br>DIA 70-84	Your blood pressure is normal.<br><br>Continue blood pressure monitoring and your current care
**LOW**	SYS 109 or less<br><br>AND<br><br>DIA 69 or less	Your blood pressure is low. Repeat once more in 5 minutes.<br><br>If you are taking blood pressure medication, contact your maternity unit within 24 hours or within 4 hours if you feel unwell (e.g. dizzy or faint).<br><br>If you are not taking medication and you are feeling well this blood pressure does not need any further action.

Figure 2: Blood Pressure Interpretation Chart for BUMP 1

165x207mm (300 x 300 DPI)

LEVEL	BLOOD PRESSURE /mmHg	ACTION
HIGH	SYS 150 or more<br>OR<br>DIA 100 or more	Your blood pressure is high<br>Sit quietly for 5 minutes then measure it again and send in the reading.<br>Contact your maternity unit for urgent assessment today (within 4 hours) and continue to monitor your BP daily.
RAISED	SYS 140-149<br>OR<br>DIA 90-99	Your blood pressure is raised<br>Sit quietly for 5 minutes then measure it again and send in the reading.<br>If your repeated reading is raised please contact your maternity unit within 24 hours and continue to monitor your BP daily.
NORMAL	SYS 110-139<br>OR<br>DIA 70-89	Your blood pressure is normal.<br>Continue blood pressure monitoring and your current care
LOW	SYS 109 or less<br>AND<br>DIA 69 or less	Your blood pressure is low. Repeat once more in 5 minutes.<br>If you are taking blood pressure medication, contact your maternity unit within 24 hours or within 4 hours if you feel unwell (e.g. dizzy or faint).<br>If you are not taking medication and you are feeling well this blood pressure does not need any further action.

Figure 3: Blood Pressure Interpretation Chart for BUMP 2

171x162mm (300 x 300 DPI)

For peer review only - http://bmjopen.bmj.com/site/about/guidelines.xhtml

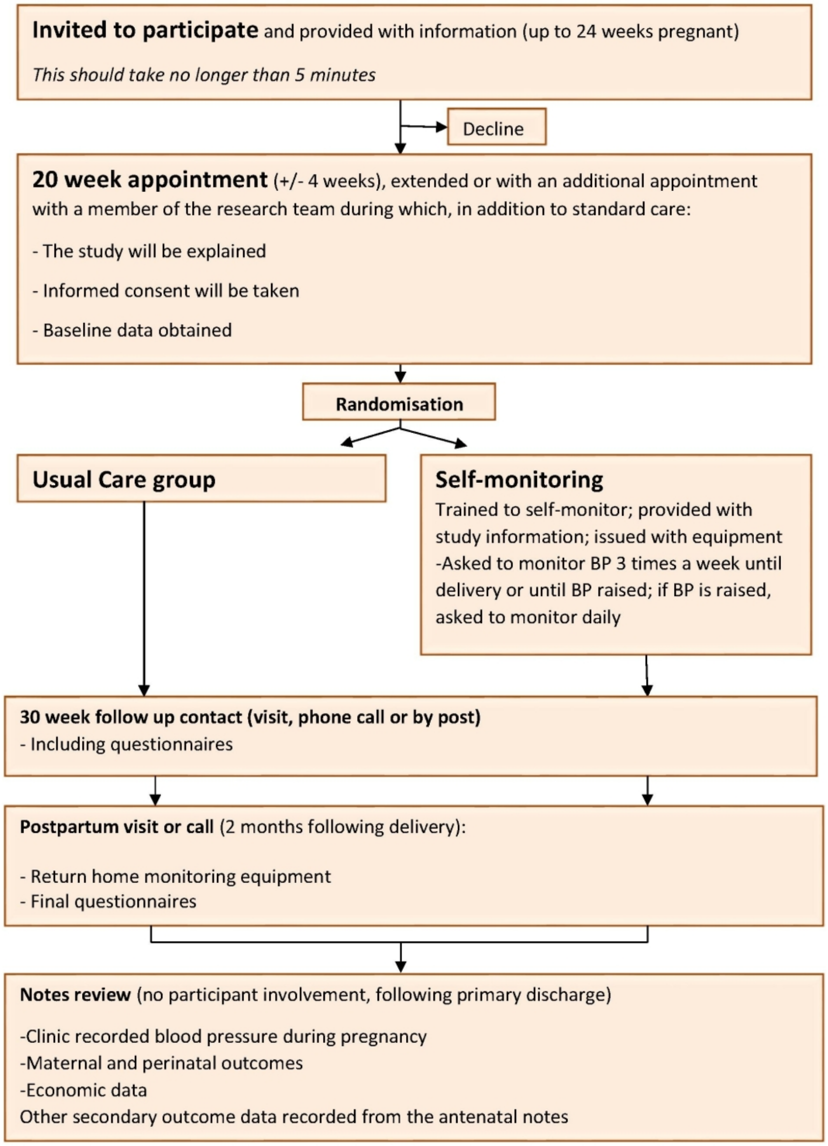

Figure 4: BUMP 1 Study Visit Chart

169x228mm (300 x 300 DPI)

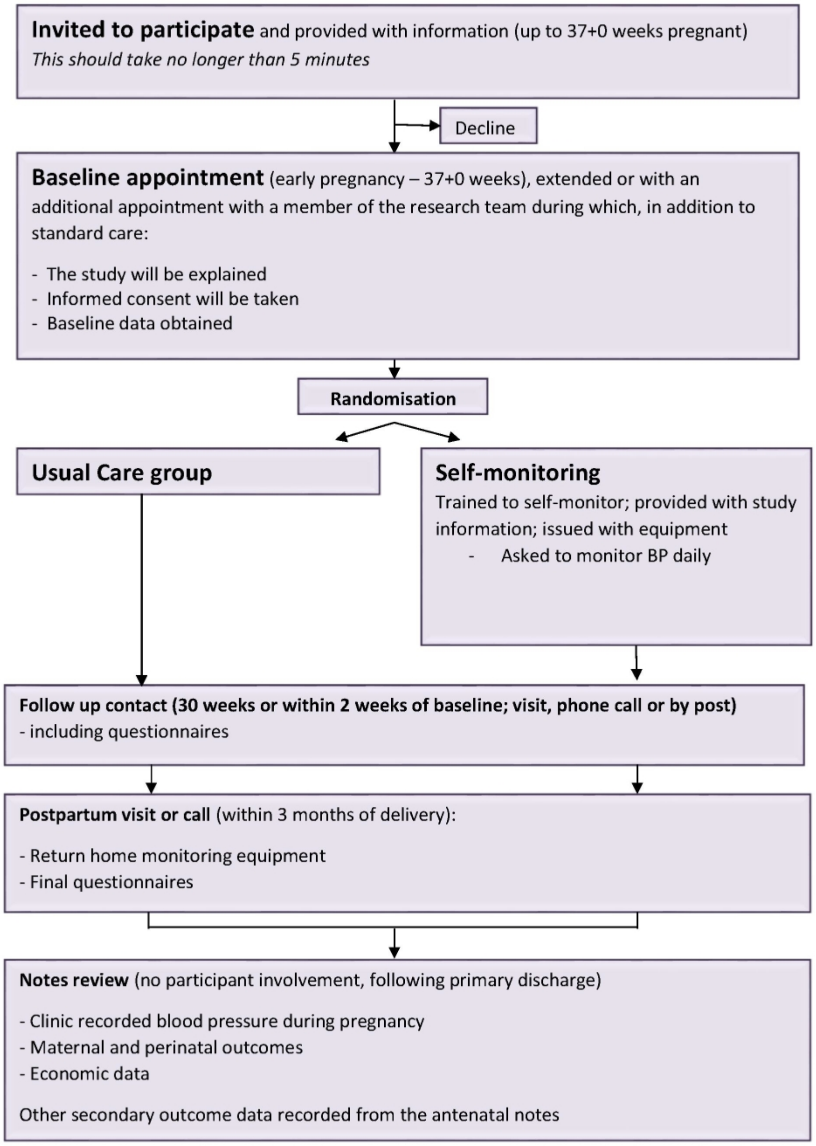

Figure 5: BUMP 2 Study Visit Chart

168x232mm (300 x 300 DPI)

STANDARD PROTOCOL ITEMS: RECOMMENDATIONS FOR INTERVENTIONAL TRIALS

SPIRIT 2013 Checklist: Recommended items to address in a clinical trial protocol and related documents*

Section/item	ItemNo	Description	Addressed on page number
**Administrative information**			
Title	1	Descriptive title identifying the study design, population, interventions, and, if applicable, trial acronym	1
Trial registration	2a	Trial identifier and registry name. If not yet registered, name of intended registry	2, 5
	2b	All items from the World Health Organization Trial Registration Data Set	NA
Protocol version	3	Date and version identifier	20
Funding	4	Sources and types of financial, material, and other support	19, 20
Roles and responsibilities	5a	Names, affiliations, and roles of protocol contributors	1, 20
	5b	Name and contact information for the trial sponsor	20
	5c	Role of study sponsor and funders, if any, in study design; collection, management, analysis, and interpretation of data; writing of the report; and the decision to submit the report for publication, including whether they will have ultimate authority over any of these activities	19, 20

For peer review only

	5d	Composition, roles, and responsibilities of the coordinating centre, steering committee, endpoint adjudication committee, data management team, and other individuals or groups overseeing the trial, if applicable (see Item 21a for data monitoring committee)	16, 19
**Introduction**			
Background and rationale	6a	Description of research question and justification for undertaking the trial, including summary of relevant studies (published and unpublished) examining benefits and harms for each intervention	4
	6b	Explanation for choice of comparators	4, 6 - 8
Objectives	7	Specific objectives or hypotheses	4
Trial design	8	Description of trial design including type of trial (eg, parallel group, crossover, factorial, single group), allocation ratio, and framework (eg, superiority, equivalence, noninferiority, exploratory)	5
**Methods: Participants, interventions, and outcomes**			
Study setting	9	Description of study settings (eg, community clinic, academic hospital) and list of countries where data will be collected. Reference to where list of study sites can be obtained	5
Eligibility criteria	10	Inclusion and exclusion criteria for participants. If applicable, eligibility criteria for study centres and individuals who will perform the interventions (eg, surgeons, psychotherapists)	5, 6
Interventions	11a	Interventions for each group with sufficient detail to allow replication, including how and when they will be administered	6 - 8

For peer review only

	11b	Criteria for discontinuing or modifying allocated interventions for a given trial participant (eg, drug dose change in response to harms, participant request, or improving/worsening disease)	8
	11c	Strategies to improve adherence to intervention protocols, and any procedures for monitoring adherence (eg, drug tablet return, laboratory tests)	7
	11d	Relevant concomitant care and interventions that are permitted or prohibited during the trial	10
Outcomes	12	Primary, secondary, and other outcomes, including the specific measurement variable (eg, systolic blood pressure), analysis metric (eg, change from baseline, final value, time to event), method of aggregation (eg, median, proportion), and time point for each outcome. Explanation of the clinical relevance of chosen efficacy and harm outcomes is strongly recommended	8, 9
Participant timeline	13	Time schedule of enrolment, interventions (including any run-ins and washouts), assessments, and visits for participants. A schematic diagram is highly recommended (see Figure)	10, 11
Sample size	14	Estimated number of participants needed to achieve study objectives and how it was determined, including clinical and statistical assumptions supporting any sample size calculations	11, 12
Recruitment	15	Strategies for achieving adequate participant enrolment to reach target sample size	12

For peer review only - http://bmjopen.bmj.com/site/about/guidelines.xhtml

Methods: Assignment of interventions (for controlled trials)

Allocation:

Sequence generation	16a	Method of generating the allocation sequence (eg, computer-generated random numbers), and list of any factors for stratification. To reduce predictability of a random sequence, details of any planned restriction (eg, blocking) should be provided in a separate document that is unavailable to those who enrol participants or assign interventions	12
Allocation concealment mechanism	16b	Mechanism of implementing the allocation sequence (eg, central telephone; sequentially numbered, opaque, sealed envelopes), describing any steps to conceal the sequence until interventions are assigned	12
Implementation	16c	Who will generate the allocation sequence, who will enrol participants, and who will assign participants to interventions	12
Blinding (masking)	17a	Who will be blinded after assignment to interventions (eg, trial participants, care providers, outcome assessors, data analysts), and how	NA
	17b	If blinded, circumstances under which unblinding is permissible, and procedure for revealing a participant's allocated intervention during the trial	NA

Methods: Data collection, management, and analysis

Data collection methods	18a	Plans for assessment and collection of outcome, baseline, and other trial data, including any related processes to promote data quality (eg, duplicate measurements, training of assessors) and a description of study instruments (eg, questionnaires, laboratory tests) along with their reliability and validity, if known. Reference to where data collection forms can be found, if not in the protocol	10, 12

For peer review only - http://bmjopen.bmj.com/site/about/guidelines.xhtml

	18b	Plans to promote participant retention and complete follow-up, including list of any outcome data to be collected for participants who discontinue or deviate from intervention protocols	7, 8
Data management	19	Plans for data entry, coding, security, and storage, including any related processes to promote data quality (eg, double data entry; range checks for data values). Reference to where details of data management procedures can be found, if not in the protocol	12
Statistical methods	20a	Statistical methods for analysing primary and secondary outcomes. Reference to where other details of the statistical analysis plan can be found, if not in the protocol	13, 14
	20b	Methods for any additional analyses (eg, subgroup and adjusted analyses)	14
	20c	Definition of analysis population relating to protocol non-adherence (eg, as randomised analysis), and any statistical methods to handle missing data (eg, multiple imputation)	13
**Methods: Monitoring**			
Data monitoring	21a	Composition of data monitoring committee (DMC); summary of its role and reporting structure; statement of whether it is independent from the sponsor and competing interests; and reference to where further details about its charter can be found, if not in the protocol. Alternatively, an explanation of why a DMC is not needed	17
	21b	Description of any interim analyses and stopping guidelines, including who will have access to these interim results and make the final decision to terminate the trial	17

Harms	22	Plans for collecting, assessing, reporting, and managing solicited and spontaneously reported adverse events and other unintended effects of trial interventions or trial conduct	17
Auditing	23	Frequency and procedures for auditing trial conduct, if any, and whether the process will be independent from investigators and the sponsor	16
**Ethics and dissemination**			
Research ethics approval	24	Plans for seeking research ethics committee/institutional review board (REC/IRB) approval	19
Protocol amendments	25	Plans for communicating important protocol modifications (eg, changes to eligibility criteria, outcomes, analyses) to relevant parties (eg, investigators, REC/IRBs, trial participants, trial registries, journals, regulators)	19
Consent or assent	26a	Who will obtain informed consent or assent from potential trial participants or authorised surrogates, and how (see Item 32)	10
	26b	Additional consent provisions for collection and use of participant data and biological specimens in ancillary studies, if applicable	15, 16
Confidentiality	27	How personal information about potential and enrolled participants will be collected, shared, and maintained in order to protect confidentiality before, during, and after the trial	18
Declaration of interests	28	Financial and other competing interests for principal investigators for the overall trial and each study site	19, 20
Access to data	29	Statement of who will have access to the final trial dataset, and disclosure of contractual agreements that limit such access for investigators	18

For peer review only

Ancillary and post-trial care	30	Provisions, if any, for ancillary and post-trial care, and for compensation to those who suffer harm from trial participation	10, 19
Dissemination policy	31a	Plans for investigators and sponsor to communicate trial results to participants, healthcare professionals, the public, and other relevant groups (eg, via publication, reporting in results databases, or other data sharing arrangements), including any publication restrictions	18
	31b	Authorship eligibility guidelines and any intended use of professional writers	20
	31c	Plans, if any, for granting public access to the full protocol, participant-level dataset, and statistical code	NA
**Appendices**			
Informed consent materials	32	Model consent form and other related documentation given to participants and authorised surrogates	NA
Biological specimens	33	Plans for collection, laboratory evaluation, and storage of biological specimens for genetic or molecular analysis in the current trial and for future use in ancillary studies, if applicable	NA

*It is strongly recommended that this checklist be read in conjunction with the SPIRIT 2013 Explanation & Elaboration for important clarification on the items. Amendments to the protocol should be tracked and dated. The SPIRIT checklist is copyrighted by the SPIRIT Group under the Creative Commons "Attribution-NonCommercial-NoDerivs 3.0 Unported" license.

For peer review only
